# Exposing the DNA methylation-responsive compartment of the leukaemic genome in T-ALL cell lines support its potential as a novel therapeutic target in T-ALL

**DOI:** 10.1186/s13148-025-01915-y

**Published:** 2025-07-03

**Authors:** Maike Bensberg, Aida Selimović-Pašić, Lisa Haglund, Júlia Goldmann, Sandra Hellberg, Colm E. Nestor

**Affiliations:** Crown Princess Victoria Children’s Hospital, Department of Biomedical and Clinical Sciences, Linköping University, Linköping, Sweden

## Abstract

T-cell acute lymphoblastic leukaemia (T-ALL) exhibits exceptionally high levels of DNA methylation, with silencing of the DNA demethylating enzyme *TET2* implicated in T-ALL’s hypermethylation phenotype. We propose that DNA hypomethylating agents (HMAs) could be particularly potent in T-ALL cells with this phenotype. Here, we used a reversible DNMT1-specific inhibitor and the conventional HMAs, 5-azacytidine and decitabine, to assess the effects of global DNA methylation loss in T-ALL cell lines and the potential of using HMAs as targeted therapeutic agents in T-ALL. We demonstrate that removal of DNA methylation, even in the absence of DNA damage, results in cell death and that toxicity is negatively correlated with methylation levels. Notably, whereas DNA demethylation caused limited transcriptional changes, key tumour suppressor genes, including *TET2*, were upregulated in a methylation-dependent manner. Few endogenous retroviruses or immune-related genes were reactivated after demethylation, challenging the contribution of ‘viral mimicry’ to HMA toxicity. Together, these findings provide fundamental insights into the role of DNA methylation in T-ALL, demonstrating that the removal of DNA methylation alone is sufficient to (i) induce cell death in T-ALL cell lines and (ii) reactivate silenced tumour suppressor genes. Our findings support the development of therapies targeting the unique methylation phenotype of T-ALL.

**Supplementary Information:**

The online version contains supplementary material available at 10.1186/s13148-025-01915-y.

## Introduction

Paediatric T-cell acute lymphoblastic leukaemia (T-ALL) is an aggressive haematological malignancy caused by the uncontrolled proliferation of immature T-cells. Whereas survival rates for T-ALL are over 85%, prognosis for relapsed T-ALL has remained poor (25–35% survival) and the intensive course of chemotherapy frequently results in serious, often fatal, side-effects [1]. Thus, development of novel therapies that prevent relapse and reduce the toxic burden of treatment is key for further improving T-ALL survival.

Altered DNA methylation patterns are a universal feature of malignancy, and DNA demethylating drugs (hypomethylating agents, HMAs) such as 5-azacytidine (AZA) and 5-aza-2′-deoxycytidine (decitabine, DAC) are routinely used in the treatment of myelodysplastic syndromes (MDS) and acute myeloid leukaemia (AML) [2–4]. Interestingly, recent studies have revealed that the T-ALL genome is uniquely characterized by exceptionally high levels of DNA methylation compared to other cancers [5–7], making it a promising candidate for HMA therapy. The hypermethylated T-ALL phenotype (~ 25% of all T-ALL patients) is frequently associated with promoter methylation and transcriptional silencing of the tumour suppressor gene and DNA demethylating enzyme, *TET2.* Notably, re-expression of *TET2* through HMA treatment resulted in increased cell death in T-ALL cells, suggesting that targeting DNA methylation may be particularly beneficial in *TET2*-silenced T-ALL [5–7].

The anti-tumour effect of HMAs has been attributed to diverse epigenetic effects including *(i)* DNA demethylation and consequent re-expression of tumour suppressor genes (TSGs) [8, 9], *(ii)* activation of the immune system by demethylation and re-expression of immune stimulatory genes [10] or *(iii)* through ‘viral mimicry’, whereby removal of DNA methylation results in expression of endogenous retroviruses (ERVs), triggering an innate or acquired anti-viral response that may clear cancer cells [11–13]. However, as AZA and DAC induce both DNA demethylation and DNA damage-associated cytotoxicity [14, 15], it has proven challenging to separate these two mechanisms and effectively assess the specific impact of global DNA demethylation on cancer cell biology. Recently, Pappalardi and colleagues developed a first-in-class reversible DNMT1-specific inhibitor, GSK-3685032 (GSK5032), which causes extensive loss of DNA methylation without activating the DNA damage response pathway [16–18], providing a powerful pharmacological tool for dissecting the molecular consequences of DNA demethylation in cancer cells.

**Fig. 1:**
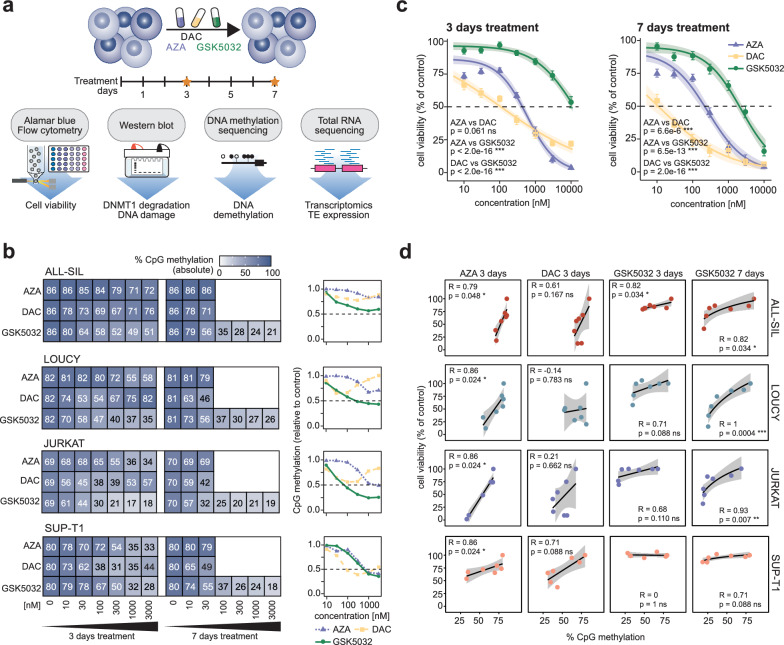
Extensive global loss of DNA methylation causes cell death. **a**, Summary of the experimental approach in this study. T-ALL cell lines were treated with 5-azacytidine (AZA), 5-aza-2′-deoxycytidine (DAC), or GSK-3685032 (GSK5032). After 3 and 7 days, the molecular response to treatment was investigated by (i) Alamar Blue assay and flow cytometry to estimate cell viability, (ii) western blot to assess DNMT1 protein degradation and the DNA damage response, (iii) whole genome DNA methylation sequencing to assess DNA demethylation and (iv) total RNA sequencing to establish transcriptomic changes including expression of transposable elements (TEs). **b**, Average global CpG methylation in four T-ALL cell lines (ALL-SIL, LOUCY, JURKAT, SUP-T1) treated with increasing concentrations of AZA, DAC, and GSK5032 for 3 or 7 days as absolute values (left) and relative to an untreated control (right). **c**, Cell viability as percentage of an untreated control for T-ALL cells treated with AZA, DAC, or GSK5032 for 3 (left) and 7 days (right). Cell viability was assessed by Alamar Blue assay. Dose–response curves as well as mean and standard error of the mean of triplicates from 11 T-ALL cell lines are shown. Statistical difference in treatment response was assessed by Wilcoxon rank-sum test. **d**, Average global CpG methylation and corresponding cell viability plotted for ALL-SIL, LOUCY, JURKAT, and SUP-T1 cells treated with AZA, DAC, or GSK5032 for 3 days or with GSK5032 for 7 days. Generalized linear regression modesl were fitted to the data as shown. Spearman’s rank correlation coefficient was used to test for correlation between cell viability and DNA methylation. Grey band depicts the 95% confidence interval of the regression model. ns (not significant) p ≥ 0.05; * p < 0.05; ** p < 0.01; *** p < 0.001

We present the first systematic comparison of the molecular, epigenetic, and transcriptional effects of AZA, DAC, and GSK5032 on T-ALL cell lines (Fig. 1A). Our findings reveal the specific cellular consequences of loss of DNA methylation in T-ALL, one of which is the reactivation of several TSGs, including *TET2* [19]. Moreover, we demonstrate that the removal of DNA methylation, even in the absence of DNA damage, is sufficient to induce cell death in T-ALL. These findings further support targeting DNA methylation as a potential therapeutic strategy for T-ALL.

## Results

### Extensive global loss of DNA methylation in the absence of DNA damage causes cell death

Considering the potential benefits of treating highly methylated T-ALL with HMAs, we aimed to systematically evaluate the DNA demethylating capacities of AZA, DAC, and GSK5032 in leukaemic cells. Initially, we characterized the DNA methylation profiles of 11 commercially available T-ALL cell lines using enzymatic methylation sequencing. Whereas assessing global DNA methylation by mass-spectrometry provides no information on the genic distribution of DNA methylation, conventional whole genome methylation sequencing is prohibitively expensive as a screening approach. Thus, we optimised ‘low-coverage whole methylome sequencing’ (average of 1% of CpGs covered) to allow for accurate determination of global 5-methylcytosine (5mC) levels as well as inform on changes in DNA methylation across distinct genomic compartments (*e.g.,* promoters, gene bodies, transposable elements) (Supplementary Fig. 1A). The concordance between global DNA methylation and methylation at long interspersed nucleotide elements (LINEs), an established proxy for global methylation levels [20], demonstrated the validity of our approach (Supplementary Fig. 1B). We identified three cell lines: LOUCY, TALL-1, and ALL-SIL, which showed higher DNA methylation levels compared to other T-ALL cell lines (*N* = 11) (Supplementary Fig. 1C, D, Supplementary Table 1, 2 and 3). Both CpG islands (CGIs), which are mostly associated with promoters, and regions outside of CGIs showed increased DNA methylation in these three cell lines (Supplementary Fig. 1E).

To evaluate the DNA demethylating activity of AZA, DAC, and GSK5032, global DNA methylation was assessed for two cell lines with high DNA methylation (ALL-SIL and LOUCY) and two additional T-ALL cell lines (JURKAT and SUP-T1) after treatment with increasing concentrations of all three drugs for 3 and 7 days with treatment being refreshed every 24 h (Supplementary Table 4, 5 and 6). Our treatment regime reflects FDA approved drug administration for AZA (daily for 7 days) and DAC (daily for 3 or 5 days). Expression of known DNA methylation-sensitive genes (*DAZL* and *GAGE12*) was detected in samples treated with all HMAs (Supplementary Fig. 2), indicating a localized transcriptional response to loss of DNA methylation. Cells treated with ≥ 100 nM GSK5032 exhibited a consistently greater loss of DNA methylation than equal doses of AZA or DAC in ALL-SIL, LOUCY and JURKAT cells (Fig. 1B). Only in SUP-T1 cells did the three HMAs show a comparable DNA demethylating response (Fig. 1B). While global CpG methylation differed in untreated cells (69%—86% CpG methylation), the lowest amount of methylation observed after treatment was more similar between the cell lines, with as little as 18–26% DNA methylation present after treatment with 3000 nM GSK5032 for 7 days (Fig. 1B). In contrast, DNA methylation at CGI promoters was almost completely removed, with only 8–15% remaining after treatment at a similar concentration and duration (Supplementary Fig. 3A, B). The rate of DNA methylation loss relative to initial CpG methylation levels was similar for all genomic locations, including ERVs and other transposable elements (TEs), when treated with AZA, DAC, or GSK5032 (Supplementary Fig. 3C). Therefore, DNA demethylation by all three HMAs seems to be a largely stochastic process occurring randomly across the genome, independently of genomic context.

Treatment with GSK5032 for 3 days resulted in only moderate toxicity, whereas long-term treatment with the drug for 7 days generated considerable cell death at high concentrations in all 11 tested T-ALL cell lines. However, GSK5032 was significantly less cytotoxic compared to AZA or DAC for both short-term (3 days) and long-term (7 days) treatment as analysed by cell viability assay and flow cytometry analysis (Fig. 1C, Supplementary Fig. 4A, B, Supplementary Fig. 5 A, B). Contrary to previous findings [21, 22], we did not detect cell cycle arrest with either treatment compared to control cells (Supplementary Fig. S5C, D), suggesting that the effects observed upon treatment with AZA, DAC or GSK5032 are likely due to cell death rather than cell cycle or growth arrest. Western blot analysis of phosphorylated H2AX (γ-H2AX), a marker for DNA damage, showed a substantial increase in JURKAT and LOUCY cells with AZA and DAC treatment but γ-H2AX could not be detected for treatment with GSK5032 (Supplementary Fig.  6A, B), confirming previous findings [16]. The DNA damage response after treatment with AZA and DAC was strikingly absent in SUP-T1 cells (Supplementary Fig.  6 C). It is likely that the observed cell death for AZA and DAC stems primarily from substantial DNA damage. In line with their mode of action [23, 24], western blot analysis showed strong loss of the DNMT1 protein with increasing concentrations of AZA and DAC, but only a slight reduction after incubation with GSK5032 in all tested cells (Supplementary Fig. 6A-C). *DNMT1* expression did not change consistently in response to treatment (Supplementary Fig. 6D), confirming loss of DNMT1 to be on the protein level.

Exploring the association between global methylation and cell viability, we found that cell toxicity was correlated to DNA methylation for 3-day treatment with AZA but not for DAC in all tested cell lines (Fig. 1D). It should be considered that DAC displayed increasing toxicity at higher concentrations (Fig. 1C) and a ‘bell-shaped’ response when analysing global DNA methylation levels with the greatest loss of methylation seen at 100–300 nM DAC at 3 days (Fig. 1B), strongly indicating that cell death through DNA damage has a major impact on the DNA methylation measurements in DAC-treated cells. Interestingly, a strong correlation was found after 7-day treatment with the DNMT1-specific inhibitor GSK5032 in ALL-SIL, LOUCY and JURKAT but not after 3 days (LOUCY and JURKAT), despite observing almost comparable levels of DNA demethylation to those at 7 days (Fig. 1D), suggesting that, in isolation of other mechanisms, only sustained DNA demethylation could impact cell viability. In contrast, loss of methylation in SUP-T1 cells after GSK5032 treatment only had a modest effect on cell viability, emphasizing a heterogenous response to DNA demethylation between cell lines. Together, the striking correlation between extensive DNA demethylation and cell viability for GSK5032 indicates that loss of DNA methylation in the absence of other contributing elements, such as DNA double-stranded breaks, results in tumour cell death.

### DNA demethylation results in limited and largely inconsistent transcriptional upregulation in leukaemic cell lines

**Fig. 2:**
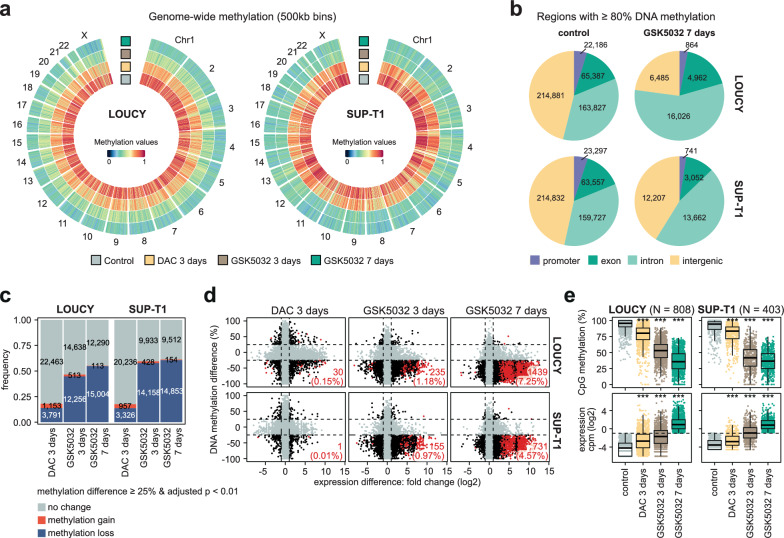
DNA demethylation results in limited and largely inconsistent transcriptional upregulation in leukaemic cells. **a**, Circos representation of genome-wide methylation levels in 500 kb bins as colour gradient in LOUCY (left) and SUP-T1 (right) cells before and after treatment with 10 nM 5-aza-2′-deoxycytidine (DAC) for 3 days or 300 nM GSK-3685032 (GSK5032) for 3 or 7 days. **b**, Pie charts visualizing the genomic location of regions (bin of ≥ 1 kb) with ≥ 80% DNA methylation in control cells only (control) or regions with ≥ 80% DNA methylation before and after treatment with GSK5032 for 7 days (GSK5032 7 days) in LOUCY and SUP-T1 cells. **c**, Number of promoters in LOUCY and SUP-T1 cells that gained or lost DNA methylation after treatment with 10 nM DAC for 3 days or 300 nM GSK5032 for 3 and 7 days. Promoters were considered to gain or lose DNA methylation with an increase or decrease of ≥ 25% DNA methylation, respectively, and an adjusted *p*-value < 0.01. **d**, Gene expression as log2 (fold change) and difference in promoter CpG methylation (%) for LOUCY and SUP-T1 cells after treatment with 10 nM DAC for 3 days or 300 nM GSK5032 for 3 and 7 days compared to an untreated control. Genes with a significant change in expression (adjusted *p*-value < 0.05 and absolute fold change > 2) and a corresponding differentially methylated promoter (adjusted *p*-value < 0.01 and absolute DNA methylation difference ≥ 25%) are marked in red with counts indicated in the bottom right corner. The dotted lines represent the thresholds for significantly up or downregulated gene expression and methylation changes. **e**, Average CpG methylation at promoters (top) and expression as log2 of counts per million (cpm) (bottom) for DNA methylation-sensitive genes identified after treatment with GSK5032 for 7 days in LOUCY and SUP-T1 cells. Wilcoxon signed-rank test; *** *p* < 0.001

To further study the direct transcriptional consequences stemming from loss of CpG methylation in T-ALL cell lines, we analysed DNA methylation and RNA expression profiles after treatment with DAC and GSK5032 from matched high-coverage enzymatic methylation sequencing and ribosomal RNA-depleted total RNA sequencing. AZA was excluded from the analysis since preferential integration of AZA into RNA prevents an accurate assessment of transcriptional changes in response to DNA demethylation as opposed to a direct effect of integration of AZA on RNA stability [25]. LOUCY, a hypermethylated cell line, and SUP-T1 cells, which showed no correlation between DNA demethylation and viability, were treated with 10 nM DAC for 3 days, or 300 nM GSK5032 for 3 and 7 days. In our previous analyses, these treatments resulted in cell viability above 50% and strong (GSK5032) to limited (DAC) loss of DNA methylation in both cell lines. All three treatments led to global loss of CpG methylation that was consistent across all chromosomes with a more pronounced reduction for GSK5032 compared to DAC (Fig. 2A, Supplementary Table 7, 8). After 7-day treatment with GSK5032, both cell lines lost more than 50% global DNA methylation, resulting in 32% and 31% genome-wide methylation in LOUCY and SUP-T1 cells, respectively.

Nevertheless, a subset of regions (bins of ≥ 1 kb) across the genome remained ≥ 80% methylated. Particularly, intronic regions retained high levels of DNA methylation in LOUCY, with a similar trend observed for SUP-T1 cells (Fig. 2B). It can be hypothesized that stable DNA methylation at promoters indicates essential silencing of gene expression by DNA methylation as no viable cells that had lost DNA methylation at these promoters could be detected. While several hundred promoters could be found in regions with ≥ 80% DNA methylation after treatment with GSK5932 for 7 days in either cell lines, the overlap between LOUCY and SUP-T1 cells was minor with only 60 promoters staying highly methylated in both (Supplementary Fig. 7A, Supplementary Table 9). Pathway analysis of genes regulated by these shared promoters showed involvement mainly in protein transport and localization (Supplementary Fig. 7B, Supplementary Table 10, 11).

While up to 50% of promoters exhibited extensive loss of at least 25% DNA methylation (adjusted *p*-value < 0.01) (Fig. 2C), only few genes were significantly upregulated (fold change > 2, adjusted *p*-value < 0.05) in response to treatment with either DAC or GSK5032 (Fig. 2D, Supplementary Fig. 7C, Supplementary Table 12). Furthermore, most upregulated genes in DAC-treated cells did not undergo DNA demethylation (Supplementary Fig. 7D), highlighting that the transcriptional changes in response to DAC are most likely independent of its DNA demethylating ability. To define a group of DNA methylation-sensitive genes that are regulated by promoter methylation, we selected genes that *(i)* were silent in untreated control cells (cpm (counts per million) < 0.5), *(ii)* were expressed and upregulated after treatment with GSK5032 for 7 days (cpm ≥ 0.5, fold change > 2, adjusted *p*-value < 0.05) and *(iii)* whose promoters were demethylated upon treatment (DNA methylation difference ≤ −25%, adjusted *p*-value < 0.01) (Supplementary Table 13). We identified 808 genes in LOUCY and 403 genes in SUP-T1 cells of which 126 were shared between the two cell lines (Supplementary Fig. 7E). These DNA methylation-sensitive genes were also demethylated and slightly upregulated after shorter treatment with both DAC and GSK5032, but most did not pass our criteria to be classified as DNA methylation-sensitive for 3-day treatments (Fig. 2E), indicating a delayed transcriptional response to loss of DNA methylation and reinforcing the notion that only prolonged removal of methylation results in discernible effects on either viability or transcription.

Together, our integration of whole genome DNA methylation and total RNA sequencing identified a small set of methylation-sensitive genes, which are regulated by DNA methylation and could play key roles in the molecular response of T-ALL to HMA treatment.

### Key tumour suppressor genes become re-expressed following DNA demethylation

**Fig. 3:**
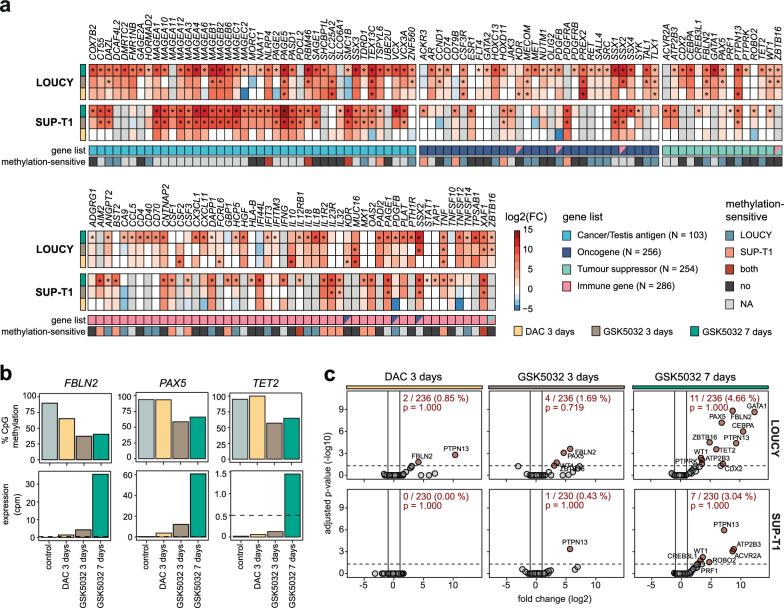
Reactivation of key tumour suppressor genes after treatment with novel DNMT1 inhibitor.** a**, Heatmaps with log2 of fold change (FC) for selected Cancer/Testis antigens, oncogenes, tumour suppressor genes and immune-related genes in LOUCY and SUP-T1 cells treated with 10 nM 5-aza-2'-deoxycytidine (DAC) for 3 days or 300 nM GSK-3695032 (GSK5032) for 3 and 7 days compared to an untreated control. Asterisks indicate statistically significant differential expression (adjusted *p*-value < 0.05). Definition as methylation-sensitive genes in LOUCY and/or SUP-T1 cells indicated below. NA, gene promoters were not covered by DNA methylation sequencing in either cell line. **b,** Average CpG methylation at promoters (top) and expression as counts per million (cpm) (bottom) for selected DNA methylation-sensitive tumour suppressor genes in LOUCY cells after treatment with 10 nM DAC for 3 days or 300 nM GSK5032 for 3 or 7 days. Dotted lines indicate cutoff for expression (cpm of 0.5). **c,** Volcano plots showing change in expression of tumour suppressor genes between treated and control cells as log2 of fold change and -log10 of the adjusted *p*-value for LOUCY (top) and SUP-T1 (bottom) cells treated with 10 nM DAC for 3 days or 300 nM GSK5032 for 3 or 7 days. Significantly up or downregulated genes shown as red dots. Total number of upregulated tumour suppressor genes and percentage of all covered genes in the gene set are written in red. *p*-values represent Benjamini Hochberg adjusted *p*-values of enrichment analysis. Lines represent thresholds for significantly upregulated genes. Dotted line: adjusted p-value < 0.05 and solid lines: fold change +/- 2

Several modes of actions have been attributed to the DNA demethylating effects of HMAs including the reactivation of TSGs. Interestingly, the T-ALL specific TSG *TET2* [5, 19], as well as *FBLN2* and *PAX5*, which have been shown to be hypermethylated and implicated in ALL malignancy [26–28], were upregulated in LOUCY cells in response to HMA treatment and were defined as methylation-sensitive (Fig. 3A, B). The T-ALL-specific oncogenes *TAL1* and *TLX1* [29] were similarly upregulated in LOUCY cells (Fig. 3A). However, there was no widespread transcriptional re-expression of TSGs or oncogenes, and neither was enriched among methylation-sensitive or upregulated genes for any treatment (Fig. 3C, Supplementary Fig. 8A-C, Supplementary Table 14).

As previously reported [30, 31], cancer/testis antigens (CTAs) were upregulated in response to DNA methylation loss upon treatment with HMAs (Fig. 3A) and were enriched among upregulated genes for short- and long-term treatment with GSK5032 but not DAC in both cell lines (Supplementary Fig. 8A-C). In accordance with increased expression of CTAs, analysis revealed enrichment for testis- and placenta-specific genes among all upregulated genes after GSK5032 treatment (Supplementary Fig. 9A). The enrichment for testis-specific genes could also be seen for methylation-sensitive genes (Supplementary Fig. 9B) and correspondingly, genes regulated by DNA methylation were predominantly involved in developmental processes (Supplementary Fig. 9C, Supplementary Table 10, 11). Interestingly, alongside differential expression of CTAs, we identified a subset of immune-related genes that were upregulated in response to treatment, with several of these genes being DNA methylation-sensitive (Fig. 3A). Moreover, immune genes were significantly enriched among all upregulated genes for treatment with GSK5032 for 7 days (Supplementary Fig. 8A-C).

Overall, several important T-ALL specific TSGs became re-expressed upon DNA demethylation along with CTAs, developmental genes, immune-related genes, and a number of oncogenes, highlighting the potential challenges associated with targeting DNA methylation globally.

### Limited transcriptional reactivation of transposable elements upon loss of DNA methylation

The re-expression of epigenetically silenced TEs in response to DNMT inhibitors has been reported to enhance anti-tumour immunity and was proposed as a potential mechanism underlying the clinical efficacy of HMAs [11, 12]. To investigate this concept further, changes in locus-specific DNA methylation and TE expression were analysed following treatment with DAC (3 days) and GSK5032 (3 and 7 days) in T-ALL cells. A general loss of DNA methylation across TEs was observed after treatment with DAC and GSK5032 in both cell lines, with a more pronounced effect for GSK5032, particularly after 7 days (Supplementary Fig. 10A), similar to global DNA demethylation. This loss of methylation was consistent across ERV, LINE, and SINE elements (Supplementary Fig. 10B, C). As expected, TEs were highly methylated (> 70% DNA methylation) in untreated cells while after 7 days of treatment with GSK5032 many TEs (range 40.6–63.9%) had become hypomethylated (< 30% methylation) (Supplementary Fig. 10B, C).

**Fig. 4:**
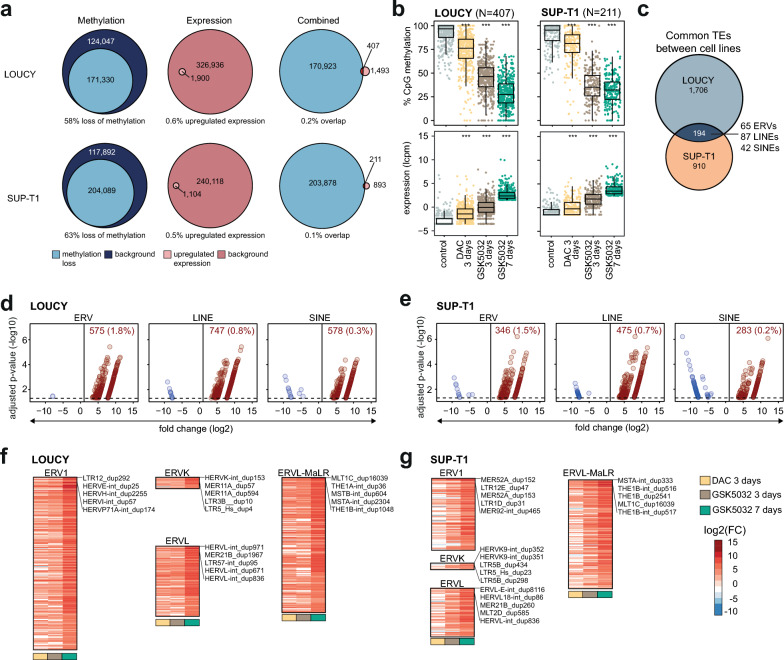
Limited transcriptional reactivation of transposable elements (TEs) upon loss of DNA methylation. **a**, Euler diagrams showing the number of TEs with significant loss of methylation (left), significant upregulation of expression (middle) and their overlap (right) in LOUCY and SUP-T1 cells treated with 300 nM GSK-3685032 (GSK5032) for 7 days. Corresponding percentages are shown below  and background represents all measured TEs for methylation and expression respectively. TEs were classified as upregulated if they exhibited a fold change > 2 and adjusted *p*-value < 0.05 and loss of methylation was defined by an adjusted p-value < 0.01 and a DNA methylation difference of ≤ −25%. **b**, Average CpG methylation of TEs (top) and expression as log_2_ of counts per million (lcpm) (bottom) for common TEs that lose methylation and gain expression in LOUCY (left) and SUP-T1 (right) cells after treatment with 5-aza-2′-deoxycytidine (DAC) or GSK5032 for 3 or 7 days. Box plot shows median with the 25th and 75th percentiles as well as the individual methylation values for all TEs. Wilcoxon signed-rank test. *** *p* < 0.001. **c,** Euler diagram showing the overlap of upregulated TEs (fold change > 2, adjusted *p*-value < 0.05) between LOUCY (blue) and SUP-T1 (orange) cells after treatment with GSK5032 for 7 days. The number of overlapping TEs assigned to ERV, LINE, and SINE elements are shown. **d, e** Volcano plots of differentially expressed TEs shown separately for ERV, LINE, and SINE elements in LOUCY (d) and SUP-T1 (e) cells treated with GSK5032 for 7 days. The number of differentially upregulated TEs and percentage out of the total number of covered TEs in every group are shown in each individual plot. Only significant TEs (adjusted *p*-value < 0.05) are shown. Red shows upregulated TEs (fold change > 2) and blue downregulated TEs (fold change < −2). **f, g** Heatmaps showing log_2_ of fold change (as compared to untreated cells) of upregulated ERVs (fold change > 2, adjusted *p*-value < 0.05) in LOUCY (f) and SUP-T1 (g) cells after treatment with DAC or GSK5032 for 3 and 7 days. ERVs shown were all differentially upregulated after GSK5032 treatment for 7 days. Names of the top 5 ERVs (based on adjusted *p*-values) are highlighted in the figure for each family. ERV, endogenous retrovirus; LINE, long interspersed nucleotide elements; SINE, short interspersed nuclear elements

After long-term GSK5032 treatment, a large portion of TEs exhibited a significant reduction in DNA methylation of at least 25% (adjusted *p*-value < 0.01), in contrast to DAC treatment, which led to a relatively modest decrease in CpG methylation (Supplementary Fig. 10D, Supplementary Table 15, 16). However, despite their marked demethylation, relatively few TEs became significantly upregulated (fold change > 2, adjusted *p*-value < 0.05) after any treatment (Supplementary Fig. 10E, Supplementary Tables 17, 18). The largest transcriptional changes were observed after 7 days of GSK5032 treatment but only 0.5—0.6% of all annotated TEs were significantly upregulated (Fig. 4A, Supplementary Fig. 10F, G, Supplementary Table 17, 18). Moreover, the transcriptional upregulation of TEs in response to HMA treatment was at most partially driven by DNA demethylating effects, as indicated by the minimal overlap between loss of DNA methylation and increased expression (< 0.2% overlap) (Fig. 4A, Supplementary Fig. 10F, G). Nevertheless, a set of TEs in both LOUCY (*n* = 407) and SUPT-1 (*n* = 211) were identified that exhibited significant methylation loss alongside increased expression following 7 days of GSK5032 treatment (Fig. 4B). These TEs were also less methylated and more expressed after shorter treatment with both DAC and GSK5032 and showed a clear pattern of higher expression with increased loss of DNA methylation, similar to the pattern observed for methylation-sensitive genes. Out of the 407 and 211 TEs that were demethylated and upregulated after treatment with GSK5032, in LOUCY and SUP-T1 cells, respectively, 386 (95%) and 171 (81%) were not expressed in untreated cells (cpm < 0.5), indicating that the removal of methylation directly facilitated the reactivation of these TEs (Supplementary Table 17, 18). However, despite the widespread demethylation of TEs following GSK5032 treatment, reactivation remained very limited, occurring in only 0.47% (147/30660) of ERVs, 0.31% (198/62298) of LINEs, and 0.05% (41/78372) of SINEs in LOUCY, and similarly in 0.15% (50/33227) of ERVs, 0.15% (104/71627) of LINEs, and 0.017% (17/99235) of SINEs in SUP-T1 cells after 7 days treatment with GSK5032.

The overall transcriptional response of TEs to 7 days GSK5032 treatment was predominantly cell line-specific, with 90% of TEs in LOUCY and 82% in SUP-T1 cells being unique to each cell line (Fig. 4C). A significantly higher proportion of ERVs was upregulated compared to both LINE and SINE elements (1.5–1.8% ERVs as compared to 0.2–0.8% LINE or SINE elements; proportion test *p* < 2.2 × 10^–16^) in both cell lines (Fig. 4D, E). Members of ERV1, ERVK, ERVL, and ERVL-MaLR were among the superfamilies with the largest number of upregulated ERVs (Fig. 4F, G) and included subfamilies such as *HERVE-int*, *HERVH-int*, *HERVL-int,* and *HERVK9-int*. Reactivation of ERVs has been suggested to lead to the induction of an anti-viral immune response through the cGAS-STING pathway and downstream activation of IFN (interferon)-α signalling [32]. We detected no enrichment of differentially expressed genes associated with the cGAS-STING pathway (LOUCY: *p* = 0.78, odds ratio (OR) = 0.68; SUP-T1: *p* = 0.59, OR = 1.17). Notably, although all genes within the cGAS-STING pathway were detected, only *NLRP4* was differentially expressed in both cell lines after 7 days of treatment (Supplementary Table 19). As expected, no downstream activation of IFN-α responses was observed in LOUCY (*p* = 0.74, OR = 0.83). While a significant enrichment of IFN-α responses was detected in SUP-T1 (*p* = 0.0007, OR = 3.11), the absence of activation of the cGAS-STING pathway—a key driver of IFN-α responses—suggests that this enrichment may reflect a general response to the treatment rather than the reactivation of a subset of ERVs (Supplementary Table 19).

Collectively, despite the extensive demethylation induced by GSK5032 across all TE classes, we observed only limited changes in their expression, with no evidence of promoting a general anti-viral response.

## Discussion

Pharmaceutical manipulation of aberrant DNA methylation patterns as a cancer treatment has been investigated for solid and haematopoietic cancers. While the hypomethylating agents AZA and DAC are currently used as treatments in AML, CMML, and MDS, unravelling the DNA methylation-specific component of their mechanism of action has proven difficult due to their DNA damage-associated effects [33]. Furthermore, the nucleoside-analogues AZA and DAC show limited bioavailability and substantial toxicity. Thus, multiple other components have been developed targeting DNA methylation or the DNA methyltransferases DNMT1 and DNMT3A/B (Supplementary Table 20). However, most newly developed drugs showed low efficacy or substantial off-target effects [34], highlighting the complex role of DNA methylation in cell biology and the potential for DNMTs to have functions that are unrelated to DNA methylation [35]. The newly developed reversible DNMT1-specific inhibitors [16] seem to be a promising advancement in the range of drugs targeting DNA methylation and provide a powerful tool for uncoupling DNA methylation from DNA damage-related toxicity, facilitating investigation of the therapeutic potential of DNA demethylation. As T-ALL has (*i*) a uniquely hypermethylated phenotype among all cancers [6], (*ii*) the hypermethylation phenotype is associated with *TET2* silencing [5], and (*iii*) *TET2* expression can be rescued with AZA [5], we investigated the potential of pharmacological removal of DNA methylation in T-ALL cell lines as an avenue for therapeutic action.

Using the DNMT1 inhibitor GSK5032, we showed that extensive loss of DNA methylation, in the absence of any DNA damage response, was significantly correlated to cell viability, providing compelling evidence for a direct cytotoxic effect of DNA demethylation. In contrast, AZA and DAC caused DNA damage and a rapid loss of DNMT1 protein, in addition to inducing DNA demethylation, thereby confounding their DNA demethylating effects. Noteworthy, a significant correlation between cell viability and methylation was only evident after 7 days of treatment with GSK5032 despite comparable DNA demethylation achieved at both 3 and 7 days, suggesting that prolonged loss of methylation is required for cell death. The transcriptional response to treatment with GSK5032 was similarly delayed implying that gene upregulation could be involved in the response to removal of methylation. Interestingly, the strongest correlation between cell viability and global DNA methylation levels was observed in the *TET2*-silenced T-ALL cell line LOUCY, where re-expression of *TET2* was also detected. We have previously shown that *TET2* is silenced through promoter methylation in 17% of primary T-ALL and that reactivation of *TET2* in *TET2*-silenced cellsoccurs in conjunction with a stronger response to AZA [5]. Furthermore, a screening of 51 T-ALL patients revealed the TSG *PAX5* to be lowly expressed and its’ promoter highly methylated [28]. While removal of DNA methylation results in transcriptional activation in only few genes, the upregulation of both *TET2* and *PAX5* after demethylation by GSK5032, as seen here, indicates that T-ALL patients with a hypermethylated phenotype could benefit from treatment with HMAs.

CTAs were significantly enriched among upregulated genes in response to DNA demethylation and, although their role in cancers remains largely unclear, in CTA-poor cancers such as leukaemia [36] reactivation of these tissue-specific antigens may contribute to promoting immunogenicity rather than tumorigenicity [37]. In line, we observed a strong upregulation of immune-related genes after 7 days of treatment with GSK5032. However, apart from CTAs and TSGs, several oncogenes, including well-characterised drivers of T-ALL [27], were upregulated in response to GSK5032. Hence, targeting DNA methylation on a global scale as a therapeutic approach must be considered with caution, as it may have both beneficial and detrimental downstream effects, which need to be carefully balanced. In contrast to GSK5032, DAC treatment for 3 days resulted in random up and downregulation of transcription that was poorly correlated with DNA methylation changes. It is possible that a longer treatment with DAC could have led to a similar effect as GSK5032. However, other studies have found gene expression changes in response to DAC to be largely independent of DNA methylation [33], calling the extent at which epigenetic mechanisms are involved in the functional effect of DAC treatment further into question.

Genome-wide removal of DNA methylation as seen for treatment with GSK5032 cannot only give rise to promoter hypomethylation and expression of previously silenced genes [8, 9, 38], but removal of intergenic DNA methylation can result in chromosomal instability [39, 40] and re-expression of ERVs [11–13]. In line with previous findings [11, 12, 16], we observed a robust demethylation of TEs after treatment with DNMT inhibitors, particularly with GSK5032, wherein almost 60% of ERVs exhibited DNA methylation loss after 7 days of treatment. However, despite this extensive demethylation, only a limited number of TEs became transcriptionally active and the majority of upregulated TEs appeared unaffected by methylation. Similarly, we did not observe substantial upregulation of genes associated with viral sensing and signalling. However, we cannot rule out that the extensive loss of methylation at TEs provides accessibility to transcription factor binding sites and other regulatory elements that could have downstream effects on immune-related processes [41].

Interestingly, SUP-T1 cells carry a pathogenic mutation in the *ATR* gene and showed no increase in γ-H2AX when treated with DAC and AZA consistent with a dysfunctional DNA damage response. Response to DNA damage is mediated by the ATM/CHK2/γ-H2AX or ATR/CHK1/γ-H2AX pathways [42–44]. However, whether the kinases ATM or ATR mediate the observed HMA-induced toxicity has not been clearly defined, as both have been associated with response to HMAs in a possibly cell-specific manner [42, 45]. The substantially reduced toxicity in SUP-T1 suggests that ATR/CHK1 may be the key effector in this context. Similarly, ATR and CHK1 have been linked to apoptosis following mitotic dysregulation induced by DAC in AML cells [46] and ATR is thought to primarily be involved in the response to replication fork stalling [47]. Based on our results, we hypothesize that ATR mediates cell death in response to AZA and DAC in SUP-T1 cells. However, SUP-T1 cells exhibited not only reduced toxicity to AZA and DAC, but also to GSK5032. This suggests that, like the acute effects of AZA and DAC, GSK5032-induced cytotoxicity may also be mediated by the DNA damage response pathway. DNMT1 has been shown to localize to double-strand breaks and contributes to the DNA damage response through interaction with the ATR effector kinase CHK1 [48, 49]. Therefore, substantial DNMT1 inhibition through DNMT inhibitors like GSK5032 may act through multiple pathways, highlighting the potential for combination therapies with DNMT inhibitors and chemotherapeutics such as PARP inhibitors.

While not being able to distinguish between epigenetic and DNA damage-related effects, both AZA and DAC have been shown to delay tumour progression and prolong disease-free survival in patient-derived xenograft mouse models of T-ALL [7, 50], showing the potential of HMAs in T-ALL treatment. Similar to our findings, loss of DNA methylation in response to DAC resulted in limited transcriptional changes in this model [50]. Additionally, GSK5032 improved survival in an AML mouse model [16]. Furthermore, the use of DAC in combination with the *BCL2* inhibitor *Venetoclax* as shown promising efficacy in individual T-ALL patients in relapse [51, 52] Indeed, there are several ongoing clinical trials investigating the combination of DAC with *Venetoclax* for high-risk and relapse/refractory T-ALL. However, the mechanism of action by which HMAs and the *BCL2* inhibitor act synergistically remains to be investigated. While there is the possibility that HMAs epigenetically prime cancer cells to respond to *Venetoclax* treatment by inducing expression of pro-apoptotic genes [53], the process could also be wholly unrelated to the DNA demethylating capacity of HMAs [54] and instead be the consequence of induction of DNA double-strand breaks and the DNA damage response by HMAs [14, 55, 56]. The mechanistic details of cancer cell death in response to combination treatment of the pro-apoptotic BCL2-specific inhibitor *Venetoclax* and HMAs remain to be investigated, possibly with the help of the novel drug GSK5032.

Our use of well-characterized T-ALL cell lines has been instrumental for dissecting the direct effects of DNA hypomethylation on leukaemic cells in a controlled setting. However, due to the nature of transformed cell lines, they may not fully capture the genetic and epigenetic diversity of primary T-ALL samples. Hypomethylating agents have shown clinical efficacy in myeloid leukaemia, and studies in primary AML cells have demonstrated that DAC-induced hypomethylation is non-random and preferentially affects hypermethylated regions [57]. While these findings highlight the therapeutic potential of targeting DNA methylation in haematological malignancies, the biological differences between AML and T-ALL limit the direct interpretation of mechanisms across these distinct cancers. Our findings provide proof-of-principle that removing DNA methylation can trigger cell death and reactivate TSGs in T-ALL cell lines, forming the basis for further preclinical and clinical studies. Confirming these results in primary T-ALL samples will be important for evaluating their clinical relevance.

In summary, we present one of the most extensive analyses of the specific effects of loss of DNA methylation on leukaemic cell lines to date. We report a significant impact of DNA methylation loss on cell viability, accompanied by re-expression of the key tumour suppressor genes like *TET2*, and find no evidence supporting the emerging paradigm of ERV reactivation as a likely mediator of the observed HMA toxicity. We provide valuable insights into the role of DNA methylation in cancer cell toxicity, highlighting its potential as a therapeutic strategy in T-ALL, particularly in highly methylated T-ALL phenotypes.

## Materials and methods

### Cell culture

T-ALL cell lines (ALL-SIL, CCRF-CEM, DND-41, HPB-ALL, JURKAT, LOUCY, MOLT-3, MOLT-4, PEER, SUPT-1, and TALL-1) were cultured in American Type Culture Collection-modified RPMI 1640 medium (Thermo Fisher Scientific, A10491). Cell culture medium was supplemented with 10% heat-inactivated foetal bovine serum (FBS) (Thermo Fisher Scientific, 11550356) and 1% penicillin–streptomycin (100 U/mL penicillin, 100 μg/mL streptomycin) (Thermo Fisher Scientific, 15140). All cells were kept in a humidified incubator at 37 °C with 5% CO_2_, passaged every 2nd or 3rd day and checked regularly for mycoplasma contamination using the MycoAlert Mycoplasma Detection Kit (Lonza, LT07-318).

### Treatment with DNA methyltransferase inhibitors

5-azacytidine (Sigma-Aldrich, A1287), 5-aza-2′-deoxycytidine (Sigma-Aldrich, A3656) and GSK-3685032 (MedChemExpress, HY-139664) were dissolved in Dimethyl Sulfoxide (DMSO) (Sigma-Aldrich, D8418) to create a stock solution of 10 mM. Single-use aliquots were kept at − 20 °C for up to 1 month. 3*10^5^ cells per millilitre were treated for 3 or 7 days with treatment being refreshed every day. Untreated control cells were similarly incubated with equivalent volumes of DMSO. Number of cells and treatment volume were scaled up or down as needed.

### Alamar Blue cell viability assay

1.5*10^5^ cells in 500 µl were treated in 24-well plates. For assessing cell viability, 90 µl cell suspension was mixed with 10 µl Alamar Blue cell viability reagent (Thermo Fisher Scientific, DAL1025) in 96-well plate, in technical triplicates, and incubated at 37 °C with 5% CO_2_ for 4 hours. Absorbance at 570 nm and 600 nm was measured on a plate reader (Tecan, Spark10M), and cell viability was calculated following the Alamar Blue reagent manufacturer's manual. All treatments were done in biological triplicates if not stated otherwise. Dose response curves were generated in R with the drc package version 3.0–1[58] fitting a three-parameter log-logistic function with the lower limit equal to 0 to the data (*drm*(fct = LL.3()). Statistical differences in response to treatment with different drugs were assessed with the Wilcoxon Rank-Sum Test, unpaired, (*pairwise.wilcox.test* from the basic stats package in R, RRID:SCR_025968) and *p*-values were adjusted for multiple testing with the Benjamini Hochberg method.

### Flow cytometry

#### Analysis of apoptosis

Following treatment with DNA methyltransferase inhibitors for 3 days, 1*10^6^ cells were counted and washed once with cold PBS (phosphate buffered saline) (Fisher Scientific, 15374875). Cells were resuspended in Annexin V binding buffer containing 0.1 M Hepes, pH 7.4 (Sigma-Aldrich, H3375), 1.4 M NaCl (Sigma-Aldrich, S9888) and 25 mM CaCl_2_ (Merck, A213887) before staining with FITC-Annexin V (Thermo Fisher Scientific, A13199), and Propidium Iodide Ready Flow Reagent (Thermo Fisher Scientific, R37169) for 15 min at room temperature. Cells were analysed on the Gallios Flow cytometer (Beckman Coulter). Compensation, gating and quantifications were done in the Kaluza analysis software version 2.1 (Beckman Coulter, RRID:SCR_016182) with the help of unstained and single stained controls.

#### Analysis of cell cycle

To analyse cell cycle, cells were fixed in 70% ethanol for 30 min at 4 °C, followed by washing with PBS and treatment with 2.5 RNase A/T1 (2 µg/µl, Thermo Fisher Scientific, EN0551) for 1 hour at 4 °C prior to Propidium Iodide staining (Thermo Fisher Scientific, P3566) for 15 min at 4 °C. Stained cells were run through the Gallios Flow cytometer (Beckman Coulter), and cell cycle was evaluated using Kaluza analysis software version 2.1 (Beckman Coulter, RRID:SCR_016182) compared to unstained control cells. Cell cycle analysis of treated and untreated cells (JURKAT and SUP-T1) was conducted in biological triplicates. Differences in cell cycle distributions over all treatments and individual treatments compared to control samples were analysed by two-sided Fisher’s exact test (*fisher.test* from the basic stats package in R, RRID:SCR_025968) with *p*-values corrected for multiple testing by Benjamini Hochberg.

### Protein extraction

10*10^6^ cells in 33 ml in T75 cell culture flasks were washed three times with cold PBS (Fisher Scientific, 15374875) and resuspended in RIPA buffer containing 150 mM NaCl (Sigma-Aldrich, S9888), 1% NP-40 (Sigma-Aldrich, 74385), 0.5% sodium deoxycholate (Sigma-Aldrich, 30970), 0.1% sodium dodecyl sulphate (Sigma-Aldrich, 71736), and 50 mM Tris pH 8.0 (VWR, E199), supplemented with 1X phosphatase (Sigma-Aldrich, 4906845001) and 1X protease inhibitor (Sigma-Aldrich, 11697498001). Cells in RIPA buffer were mixed well and incubated on ice for 30 min before centrifugation at 8000xg for 10 min at 4 °C. Supernatant was transferred to a fresh Eppendorf tube and protein concentration was determined with the BCA protein assay kit (Thermo Fisher Scientific, 23235) following manufacturer's instructions.

### Western Blot

20 μg of proteins were lysed with 2 × Laemmli buffer (Bio-Rad, 1610737) at 60 °C for 15 min. Proteins were separated on 4–15% Mini-PROTEAN TGX Stain-Free Precast Gels (Bio-Rad, 4568086) and transferred to Trans-Blot Turbo Midi 0.2 μm PVDF membranes (Bio-Rad, 1704157) using the Trans-Blot Turbo Transfer System (Bio-Rad). Membranes were blocked for 1 hour with 5% non-fat dry milk in TBS-T (20 mM Tris base, 150 mM NaCl) supplemented with 0.1% Tween (Bio-Rad, 1706531) for 1 hour. After blocking, the membranes were incubated with primary antibodies against DNMT1 (1:1000, Cell Signalling Technology, 5032, RRID:AB_10548197), Phospho-Histone H2A.X (1:1000, Cell Signalling Technology, 9718) or β-actin (1:1000, Cell Signalling Technology, 8457, RRID:AB_2118009) in 5% non-fat dry milk in TBS-T overnight. After three washes in TBS-T, membranes were incubated with goat anti-Rabbit or anti-Mouse IgG (H + L)-HRP-conjugated secondary antibody (1:3000, Bio-Rad, 1706515, 1706516, RRID:AB_11125142, RRID:AB_2921252) in 5% Bovine Serum Albumin (Roche, 03117332001) in TBS-T for 1 hour at room temperature. Membranes were imaged with Clarity Western ECL Substrates (Bio-Rad, 1705060) using the ChemiDoc MP imaging system (Bio-Rad).

### DNA and RNA isolation

DNA and RNA was extracted using the Quick-DNA/RNA Kit (Zymo Research, D7001 and D7003). The manufacturer’s instructions were followed with the addition of in-column treatment with RNase A/T1 (Thermo Fisher Scientific, EN0551) and DNase I (Zymo Research, E1010) for DNA and RNA samples, respectively. After binding of DNA/RNA to the extraction columns, DNA was incubated with 4U RNase A/T1 and RNA was treated with 5U DNase I for 15 min at room temperature. DNA and RNA were quantified using a NanoDrop 2000 spectrophotometer (Thermo Fisher Scientific).

### Quantitative PCR

200 ng RNA was converted into cDNA using the High-Capacity cDNA Reverse Transcription Kit (Applied Biosystems, 4368814), according to the manufacturer’s instructions. Samples were incubated at 37 °C for 2 hours followed by inactivation for 5 min at 85 °C. cDNA was diluted 1:10 to a total volume of 200 μl. 4.5 μl of cDNA was mixed with 5 µl of TaqMan Fast Universal PCR Master Mix (Thermo Fisher Scientific, 4352042) and 0.5 µl TaqMan probes (Thermo Fisher Scientific): DNMT1 (Hs00945875_m1), DNMT3A (Hs01027166_m1), DNMT3B (Hs00171876_m1), DAZL (Hs00154706_ml), GAGE12 (Hs04190947_gH), and GAPDH (Hs02758991_g1). qPCR was carried out in technical duplicates on a QuantStudio 7 Flex Real-Time PCR System (Thermo Fisher Scientific) with the temperature profile of: 95 °C for 20 s and 40 cycles of 95 °C for 1 s and 60 °C for 20 s. Expression of target genes was quantified using ΔCt method normalized to *GAPDH* expression.

### Enzymatic Methyl-sequencing library preparation

200 ng DNA was diluted in 0.1X TE with 0.001 ng methylated pUC19 control DNA and 0.02 ng unmethylated lambda control DNA to a total volume of 50 µl. DNA was fragmented by sonication (Bioruptor, Diagenode) to an average fragment length of 290 bp. Sequencing libraries were prepared using the NEBNext Enzymatic Methyl-seq Kit (New England Biolabs, E71207S/L) following the manufacturer’s instructions for standard library inserts. In short, after end-prep of the DNA, sequencing adaptors were ligated to the fragmented DNA. Following, methylated and hydroxy-methylated cytosines were oxidized by TET2 and the DNA was denatured by incubation with formamide at 85 °C for 10 min. Cytosines were deaminated by the APOBEC enzyme and libraries were PCR amplified for 4 cycles. Sequencing libraries were quantified using the Qubit 1X dsDNA High Sensitivity assay (Thermo Fisher Scientific, Q33231) and library size was estimated on a Bioanalyzer with the high sensitivity DNA assay (Agilent, 5067–4626). Libraries were sequenced on a NextSeq2000 or MiSeq machine (Illumina) with 150 or 250 bases paired-end reads. For low-coverage whole methylome sequencing > 100,000 read pairs were sequenced and deep EM-seq resulted in an average of 115 million read pairs per sample. To ensure successful library conversion, only libraries with < 2% DNA methylation at CHG and CHH contexts were considered for analysis.

### Total RNA sequencing library preparation

Quality of RNA for next generation sequencing was evaluated on the Agilent Bioanalyzer (RRID:SCR_018043) using the RNA 6000 Nano kit (Agilent, 5067–1511) with an average RIN of 9.0. RNA sequencing libraries were only prepared from RNA with RIN > 7. Directional rRNA-depleted total RNA sequencing libraries were prepared from 500 ng RNA using the NEBNext rRNA Depletion Kit v2 (New England Biolabs, E7405L), NEBNext Ultra II Directional RNA Library Prep (New England Biolabs, E7765S), and NEBNext Multiplex Oligos for Illumina (New England Biolabs, E7335S) according to the manufacturer’s instructions with slight adjustments as follows. After removal of ribosomal RNA (rRNA) using DNA probes and digestion with RNase H and DNase I, RNA was fragmented for 10 minutes at 94 °C for an average library insert size of 300 nucleotides. First strand cDNA synthesis was performed for 10 minutes at 25 °C, 50 minutes at 42 °C and 15 minutes at 70 °C. Libraries were PCR amplified for 9 cycles. Sequencing libraries were quantified using the Qubit 1X dsDNA High Sensitivity assay (Thermo Fisher Scientific, Q33231), and library size was estimated on a Bioanalyzer with the high sensitivity DNA assay (Agilent, 5067–4626); average sequencing library size: 360 bp. Libraries were sequenced on a NextSeq2000 machine (Illumina) with 150 bases sequenced from each end (paired-end). The median sequencing depth was 102 million reads per sample (min: 85 million and max: 725 million reads).

### Processing of Enzymatic Methyl-sequencing data

Raw sequencing reads were trimmed with Trim Galore version 0.6.10 (https://github.com/FelixKrueger/TrimGalore; RRID:SCR_011847) using default parameters including *–paired* and *–fastqc*. Sequences for pUC19 (M77789.2) and lambda (J02459.1) were added to the human hg38 reference genome (GCA_000001405.15_GRCh38_no_alt_analysis_set). Trimmed reads were aligned (bwameth.py) to the indexed genome (*bwameth.py index*) with bwa-meth version 0.2.7 (https://github.com/brentp/bwa-meth arXiv:1401.1129v2) with default parameters. Using samtools version 1.13 [59] (RRID:SCR_002105), aligned reads were converted to bam format (*samtools view*) and sorted (*samtools sort*). Duplicate reads were marked with Picard *MarkDuplicates* (http://broadinstitute.github.io/picard; RRID:SCR_006525) with default parameters and files were indexed with *samtools index*.

DNA methylation was called with *MethylDackel extract* (version 0.6.1, https://github.com/dpryan79/MethylDackel; RRID:SCR_025850) trimming reads by five bases from each end using the flags *–nOT 6,5,6,5* and *–nOB 6,5,6,5*. Non-CpG methylation was extracted by including the flags *–CHG* and *–CHH*. For CpG methylation calls, strand-specific information was merged (*MethylDackel extract –mergeContext –nOT 6,5,6,5 –nOB 6,5,6,5*). Additionally, the number of reads covering each CpG was extracted with *MethylDackel extract (–nOT 6,5,6,5; –nOB 6,5,6,5; –mergeContext, –counts*).

#### Analysis of low-coverage whole methylome sequencing

From the strand-merged CpG methylation calls, global average CpG methylation levels were calculated after excluding both control DNAs (pUC19: M77789.2; lambda: J02459.1) and mitochondrial DNA (chrM). *Bedtools intersect* from the BEDTools suit of functions version 2.26.0 [60] (RRID:SCR_006646) was used to intersect strand-merged CpG methylation calls with regions of interest to calculate average region-specific CpG methylation. Annotation of CpG islands (CGIs), genes, ERVs, LINEs, and SINEs for hg38 were downloaded from the UCSC table browser. Promoters were defined at starting 1 kb upstream and ending 500 bp downstream of transcription start sites. Promoters were considered CGI promoters if 20% of the promoter overlapped with a CGI or 50% of the CGI overlapped with the promoter.

#### Analysis of high-coverage Enzymatic Methyl-sequencing

For high-coverage DNA methylation sequencing (matched samples with total RNA sequencing), DNA methylation at CpGs was called with a minimum coverage of five reads per CpG (MethylDackel extract -d 5 *–mergeContext –nOT 6,5,6,5 –nOB 6,5,6,5*). For further analysis, methylKit compatible DNA methylation calls were extracted with MethylDackel extract *–nOT 6,5,6,5 –nOB 6,5,6,5 –methylKit*. Downstream analysis was conducted in R (version 4.2.0) using methylKit version 1.27.1 [61] (RRID:SCR_005177). CpG methylation calls with a minimum coverage of five reads were imported (*methRead*). CpG methylation at regions of interest was summarized (*regionCounts*) and normalized (*normalizeCoverage*) in groups separated by cell line. Annotation of CpG islands (CGIs), genes, and TEs, based on TElocal and Repeatmasker (RRID:SCR_012954), for hg38 were downloaded from the UCSC table browser. Strand-specific DNA methylation was merged to a CpG dinucleotide context when uniting samples by adding the parameter destrand = TRUE. Differential DNA methylation was determined by Fisher’s exact test and *p*-values were adjusted for multiple testing using Benjamini Hochberg (*calculateDiffMeth(test* = *"fast.fisher", slim* = *FALSE, adjust* = *"BH")).*

To identify regions retaining DNA methylation, methylation calls were grouped across the genome in bins of 1000 base pairs, overlapping by 500 base (*methylKit::tileMethylCounts* with win.size = 1000 and step.size = 500), normalized (*methylKit::normalizeCoverage*) and united (*methylKit::unite*) including only regions covered in control samples and samples treated with GSK5032 for 7 days within each cell line. Regions with ≥ 80% DNA methylation in control and treated samples were selected and overlapping or adjacent regions were merged.

Genomic features were annotated using the genomation package (version 1.30.0; RRID:SCR_003435) and only NM_* and NR_* transcripts were included for further analysis. Promoters were defined as being located 1000 base pairs upstream to 500 base pairs downstream from a transcription start sites.

### Processing of total RNA sequencing data

Raw RNA reads (fastq) were trimmed with Trim Galore (version 0.6.10; https://github.com/FelixKrueger/TrimGalore; RRID:SCR_011847) using default parameters including removing Illumina adapters (AGATCGGAAGAGC) and bad quality bases (Phred score 20). Paired-end reads were aligned to the human reference genome (hg38 assembly GCA_000001405.15) with Spliced Transcripts Alignment to a Reference (STAR; version 2.5.2b; RRID:SCR_004463) [62]. For more reliable quantification of TEs, STAR was run with the following parameters: –outFilterMultimapNmax 500 –winAnchorMultimapNmax 500, to increase the number of reported multi-mapped reads. Additional parameters used for the alignment were based on the ENCODE recommendations for RNA-seq alignment and included: –alignSJoverhangMin 8, –alignSJDBoverhangMin 1, –alignSJDBoverhangMin 1, –outFilterMismatchNoverReadLmax 0.04, –alignIntronMin 20, –alignIntronMax 1000000, –alignMatesGapMax 1000000, –outFilterType BySJout, –outSAMattributes NH HI AS NM MD, –outSAMstrandField intronMotif and –sjdbScore 1. Gene quantification and locus-specific TE quantification was performed with TElocal (https://github.com/mhammell-laboratory/TElocal; version 1.1.1; RRID:SCR_023208) from the TEToolkit suite (https://hammelllab.labsites.cshl.edu/software) [63] with the following flags: –mode multi and –stranded reverse.

Raw counts were analysed in R (version 4.3.1) with the edgeR package [64] (version 4.0.16; RRID:SCR_012802). The two cell lines were processed independently. For each cell line, genes and TEs were analysed separately. rRNA genes were removed from the raw counts prior to downstream analysis. TEs categorized as LTR, ERV, L1, L2 and Alu, and MIR were selected for the downstream analysis of TEs. Lowly expressed genes/TEs were filtered out using *filterByExpr* using the default parameters. Counts-per-million (cpm) and log2cpm were calculated using the *cpm* function. Statistical differences between the samples were determined using *exactTest* comparing treated to untreated cells for each respective cell line (Treated–Untreated). The dispersion was set to 0.16 (biological coefficient of variation (bcv; square-root dispersion)) = 0.4) when calculating genewise exact tests as recommended for well-controlled human experimental data in edgeR [64]. *p*-values were corrected for multiple testing with Benjamini–Hochberg using *p.adjust* (package stats version 4.3.1).

Genes and TEs were categorized as upregulated (adjusted *p*-value < 0.05 and fold change (FC) > 2 (log_2_FC > 1)), downregulated (adjusted *p*-value < 0.05 and FC < −2 (log2FC < −1), or with no significant change. Genes were defined as DNA methylation-sensitive based on (i) cpm < 0.5 in untreated cells, (ii) cpm ≥ 0.5, fold change > 2 upon treatment with GSK5032 (adjusted *p*-value < 0.05), and (iii) DNA methylation difference ≤ −25% as compared to untreated cells (adjusted *p*-value < 0.01).

### Enrichment analysis

Enrichment analysis was performed to check for enrichment of one of the following gene sets: (1) immune-related genes, (2) tumour suppressor genes (TSGs), (3) oncogenes, and (4) cancer/testis antigens (CTAs) among the upregulated genes (fold change > 2, adjusted *p*-value < 0.05) and the DNA methylation-sensitive genes. The enrichment analysis was performed for each cell line separately using a hypergeometric test in R (basic stats package): phyper (q-1, m, n, k, lower.tail = F) with:

- q: number of genes in the gene set that are upregulated/methylation-sensitive
- m: number of genes in the gene set that are covered by RNA-seq/whose promoters lose DNA methylation (DNA methylation difference ≤ −25% as compared to untreated cells (adjusted *p*-value < 0.01))
- n: number of genes that are covered by RNA-seq/whose promoters lose DNA methylation but are not part of the gene set
- k: total number of genes that are upregulated/methylation-sensitive

Immune-related genes involved in anti-viral immune responses as well as the previously identified AZA immune gene signature [10] were selected for the enrichment analysis of immune genes. Genes were defined as TSGs or oncogenes based on the annotation from the Network of Cancer Genes and Healthy Drivers [65]. Genes were defined as CTAs based on Carter et al. [66]. All gene sets used for enrichment analyses can be found in **Supplementary Table 14**. Two hundred random genes that were covered by sequencing were included as a negative control. Enrichment analysis was performed for 1000 iterations of random gene sets as described above.

The molecular signature database was used to specifically test for the enrichment of the hallmark signature dataset HALLMARK_INTERFERON_ALPHA_RESPONSE (M5911) and the CP:REACTOME dataset REACTOME_STING_MEDIATED_INDUCTION_OF_HOST_IMMUNE_RESPONSES (M27045) against number of genes that are covered by RNA-seq, using one-sided Fisher's exact test, implemented by the R function *fisher.test* selecting the option *alternative* = *greater*. For tissue-specific gene enrichment, *teEnrichment* from the R package TissueEnrich (version 1.22.0) [67] was used with the Human Protein Atlas [68] as reference data set for tissue enrichment defined using the option *rnaSeqDataset* = *1*. Only genes that were tissue-enriched (*tissueSpecificGeneType* = *2*) were included. Tissues with an adjusted *p*-value ≤ 0.05 were considered significantly enriched.

### Pathway analysis

Pathway enrichment analysis based on Gene Ontology (GO) was performed using *enrichGO* from clusterProfiler (version 4.10.1; RRID:SCR_016884) [69]. Depending on the list of input genes the background was either corrected for all genes losing methylation (≥ 25% mean methylation difference as compared to untreated cells, adjusted *p*-value < 0.01) for the methylation-sensitive genes or all promoters overlapping regions with 80% methylation in untreated cells for promoters retaining methylation after treatment with GSK5032 for 7 days. Pathways with an adjusted *p*-value ≤ 0.05 were considered significant. Enriched pathways were visualized using the *dotplot* function from clusterProfiler.

### Data visualization

Data were visualized using GraphPad Prism 10 (version 10.0.2; RRID:SCR_002798) or the R packages circlize (function *circos.heatmap* for circos plots; version 0.4.16; RRID:SCR_002141), ggplot2 (for violin, bar, and box plots; version 3.5.1; RRID:SCR_014601), pheatmap (for heatmaps; version 1.0.12; RRID:SCR_016418), and eulerr (for proportional Venn diagrams; version 7.0.2; RRID:SCR_022753).

### Statistics and reproducibility

Statistical analysis was conducted in R as described if not stated otherwise.

## Supplementary Information

Additional file 1. Supplementary Fig. 1.**a**, Overview of our low-coverage whole methylome sequencing approach. DNA was extracted and base pair resolution DNA methylation libraries were prepared by enzymatic methyl-conversion using TET2 and APOBEC. Sequencing libraries were sequenced at low coverage and DNA methylation per covered CpG was assessed from the number of sequenced unconverted (methylated) cytosines (C) and the number of sequenced thymines (T) representing unmethylated cytosine. Global or region-specific DNA methylation was determined by calculating the average across all covered CpGs.**b**, Average global CpG methylation assessed by low-coverage whole methylome sequencing (black) and DNA methylation at long interspersed nucleotide elements (LINE) (grey) for T-ALL cell lines. Individual points for each replicate (left) and mean of duplicates (right).**c,d** Average CpG methylation as percentage genome-wide (global) or at CpG islands for 11 T-ALL cell lines assessed by low-coverage whole methylome sequencing. Individual replicates (c) and mean of duplicates (d).** e,** Percentage of CpG methylation relative to the average over all cell lines for CpG islands and globally. .
Supplementary Fig. 2. Expression of genes known to get re-expressed upon DNA demethylation, *DAZL*, and *GAGE12*, after treatment with increasing concentrations of 5-azacytidine (AZA), 5-aza-2′-deoxycytidine (DAC), and GSK-3685032 (GSK5032) for 3 (left) and 7 days (right). Expression determined by qPCR and shown relative to housekeeping gene *GAPDH*. Cross indicates that no expression was detected by qPCR.
Supplementary Fig. 3.**a**, Average global CpG methylation relative to an untreated control across four T-ALL cell lines divided based on genomic location. Cells were treated with increasing concentrations of 5-azacytidine (AZA), 5-aza-2′-deoxycytidine (DAC), and GSK-3685032 (GSK5032) for 3 or 7 days. **b,** Average absolute CpG methylation across four T-ALL cell lines treated with 3000 nM GSK5032 for 7 days divided based on genomic location. **c**, Average global CpG methylation relative to an untreated control across four T-ALL cell lines treated with increasing concentrations of AZA, DAC and GSK5032 for 3 or 7 days divided based on genomic location. SINE, short interspersed nuclear elements; LINE, long interspersed nucleotide elements; ERV, endogenous retrovirus; CGI, CpG island.
Supplementary Fig. 4. **a, b,** Cell viability as percentage of untreated control for eleven T-ALL cells treated with 5-azacytidine (AZA), 5-aza-2′-deoxycytidine (DAC), or GSK-3685032 (GSK5032) for 3 (a) and 7 days (b). Cell viability assessed by Alamar Blue assay in triplicates. Dose-response curves and mean and standard error of the mean of triplicates from eleven T-ALL cell lines are shown. Statistical difference in treatment response for all cell lines combined per duration of treatment was assessed by Wilcoxon Rank Sum Test.
Supplementary Fig. 5. **a**, Flow cytometry analysis after staining with propidium iodide (PI) and Annexin V of T-ALL cell lines treated with indicated concentrations of 5-azacytidine (AZA), 5-aza-2′-deoxycytidine (DAC), and GSK5-3685032 (GSK5032) for 3 days. Percentages of alive cells (PI-/Annexin V-, green), apoptotic cells (PI-/Annexin V+, blue), and necrotic cells (PI+/Annexin V+, red) are indicated. **b,** Percentage of dead cells (apoptotic and necrotic) after treatment of JURKAT and SUP-T1 cells with indicated concentrations of AZA, DAC, and GSK5032 for 3 days. Mean and standard deviation of biological triplicates shown. Based on flow cytometry analysis of cells stained with PI and Annexin V (examples shown in panel a). c, Cell cycle analysis with PI staining in JURKAT and SUP-T1 cells treated with indicated concentrations of AZA, DAC, and GSK5032 for 3 days. Gating strategy shown for one representative replicate. G1-phase: blue, G2/M-phase: red; S-phase: green. d, Percentage of cells in each phase of the cell cycle based on PI staining for JURKAT and SUP-T1 cells treated with indicated concentrations of AZA, DAC, and GSK5032 for 3 days. Mean of biological triplicates shown. Fisher’s exact test with p-values adjusted for multiple testing using Benjamini Hochberg. ns, adjusted p-values > 0.05.
Supplementary Fig. 6. **a-c, **Western blot of DNMT1, phosphorylated H2AX (γ-H2AX), and β-actin (BACTIN) for JURKAT (a), LOUCY (b), and SUP-T1 (**c**) cells treated with increasing concentrations of 5-azacytidine (AZA), 5-aza-2′-deoxycytidine (DAC,) and GSK-3685032 (GSK5032) for 3 days. For SUP-T1, protein extracted from untreated JURKAT cells (-AZA) and JURKAT cells treated with 1000 nM AZA (+AZA) were included as antibody controls. **d**, Expression of DNA methyltransferases *DNMT1*, *DNMT3A*, and *DNMT3B* after treatment with increasing concentrations of AZA, DAC, and GSK5032 for 3 (top) and 7 days (bottom). Expression determined by qPCR and shown relative to housekeeping gene *GAPDH*.
Supplementary Fig. 7. **a**, Venn diagram showing the overlap between genes whose promoters keep ≥ 80% DNA methylation after treatment with 300 nM GSK-3685032 (GSK5032) for 7 days in LOUCY (blue) and SUP-T1 cells (red). **b**, Pathway enrichment analysis for the 60 genes whose promoters retain ≥ 80% DNA methylation after treatment with 300 nM GSK5032 in both LOUCY and SUP-T1 cells (overlap shown in panel a). List of genes can be found in Supplementary Table 9. **c**, Number of genes in LOUCY and SUP-T1 cells that are up or downregulated after treatment with 10 nM 5-aza-2′-deoxycytidine (DAC) for 3 days or 300 nM GSK5032 for 3 and 7 days. Genes were considered downregulated with a fold change (FC) < -2 and an adjusted p-value < 0.05. Genes were considered upregulated with FC > 2 and an adjusted p-value < 0.05. Otherwise, the expression of genes was considered to not change. **d**, Euler plot showing the overlap between upregulated genes (FC > 2 and adjusted p-value < 0.05) in red and promoters that lost DNA methylation (DNA methylation difference ≥ 25% and adjusted p-value < 0.01) in blue in LOUCY and SUP-T1 cells treated with 10 nM DAC for 3 days or 300 nM GSK5032 for 3 and 7 days. **e**, Venn diagram showing the overlap between methylation-sensitive genes defined after treatment with 300 nM GSK5032 for 7 days in LOUCY (blue) and SUP-T1 cells (red). Definition of methylation-sensitivity for each analysed gene can be found in Supplementary Table 13.
Supplementary Fig. 8. **a**, -log10 of the adjusted p-value for enrichment analysis (phyper) of immune-related genes, cancer/testis antigens, tumour suppressors, and oncogenes among upregulated genes in LOUCY and SUP-T1 cells after treatment with 10 nM 5-aza-2′-deoxycytidine (DAC) for 3 days or 300 nM GSK-3685032 (GSK5032) for 3 or 7 days and genes defined as methylation-sensitive after treatment with 300 nM GSK5032 for 7 days. Genes included in each gene list can be found in Supplementary Table 14. **b, c,** Volcano plots showing change in expression of selected gene sets between treated and control cells as log2 of fold change and -log10 of the adjusted p-value for LOUCY (b) and SUP-T1 (c) cells treated with 10 nM DAC for 3 days or 300 nM GSK5032 for 3 or 7 days. Significantly up or downregulated genes shown as red dots. Total number of upregulated genes and percentage of all covered genes in gene set written in red. Written p-values represent Benjamini Hochberg adjusted p-values of enrichment analysis.
Supplementary Fig. 9. **a,** -log10 of the adjusted p-value for tissue enrichment analysis on upregulated genes in LOUCY and SUP-T1 cells after treatment 300 nM GSK-3685032 (GSK5032) for 3 or 7 days. **b**, -log10 of the adjusted p-value for tissue enrichment analysis on methylation-sensitive genes in LOUCY and SUP-T1 cells defined after treatment 300 nM GSK5032 for 7 days. Definition of methylation-sensitivity for each analysed gene can be found in Supplementary Table 13. **c**, Pathway enrichment analysis for methylation-sensitive genes defined after treatment with 300 nM GSK5032 for 7 days in LOUCY (left) and SUP-T1 cells (right). Pathways related to tissue development are highlighted in bold. Definition of methylation-sensitivity for each analysed gene can be found in Supplementary Table 13.
Supplementary Fig. 10. **a,** Circos representation of genome-wide methylation levels of transposable elements (TEs; including ERV, LINE, and SINE) in LOUCY (left) and SUP-T1 (right) cells before and after treatment with 10 nM 5-aza-2′-deoxycytidine (DAC) for 3 days or 300 nM GSK-3685032 (GSK5032) for 3 or 7 days. Average level of methylation of all TEs in 500kb windows is shown and displayed by chromosome. The colour gradient indicates DNA methylation level. **b, c,** Violin plots of DNA methylation levels for LOUCY (b) and SUP-T1 (c) cells untreated and treated with 10 nM DAC for 3 days or 300 nM GSK5032 for 3 or 7 days. Percentages of TEs within each class (ERV, LINE, and SINE) that had methylation levels below 0.3 or above 0.7 are shown on top. Wilcoxon signed-ranked test; *** p < 0.001. **d**, Bar graphs of percent of ERV, LINE and SINEs that showed no change (grey), loss (blue), or gain of methylation (red) after treatment with DAC and GSK5032 in LOUCY (left) and SUP-T1 (right) cells. Loss and gain of methylation were based on a methylation difference of ± 25% (compared to untreated) and adjusted p-value < 0.01. Percent of TEs gaining methylation was below 0.4% in both LOUCY and SUP-T1 for all treatments. **e**, Number of differentially expressed ERV, LINEs, and SINEs in LOUCY (left) and SUP-T1 (right) cells that were upregulated (red; fold change > 2, adjusted p-value < 0.05) or downregulated (blue; fold change < -2, adjusted p-value < 0.05) after treatment with 10 nM DAC for 3 days or 300 nM GSK5032 for 3 or 7 days compared to untreated cells. Total number of TEs detected are depicted on top of each graph for each cell line, respectively. **f,g**, Euler diagrams showing the number of TEs with significant loss of methylation (left), significant upregulation of expression (middle), and their overlap (right) in LOUCY and SUP-T1 cells treated with 10 nM DAC (f) or 300 nM GSK5032 (g) for 3 days. Corresponding percentages are shown below and background represents all measured TEs. TEs were classified as upregulated if they exhibited a fold change > 2 and adjusted p-value < 0.05 and loss of methylation was defined as TEs with an adjusted p-value < 0.01 and a DNA methylation difference of ≤ -25%.

Additional file 2. Supplementary Table 1. Sequencing metrics for low pass whole methylome sequencing of untreated T-ALL cell lines.
Supplementary Table 2. Average CpG methylation as percentage, globally and at specific genomic regions, assessed by low pass whole methylome sequencing of untreated T-ALL cell lines.
Supplementary Table 3. Average coverage per CpG for low pass whole methylome sequencing of untreated T-ALL cell lines.
Supplementary Table 4. Sequencing metrics for low pass whole methylome sequencing of ALL-SIL, LOUCY, JURKAT and SUP-T1 cells treated with increasing concentrations of 5-azacytidine (AZA), 5-aza-2′-deoxycytidine (DAC), and GSK-3685032 (GSK5032) for 3 and 7 days.
Supplementary Table 5. Average CpG methylation as percentage, globally and at specific genomic regions, assessed by low pass whole methylome sequencing of ALL-SIL, LOUCY, JURKAT, and SUP-T1 cells treated with increasing concentrations of 5-azacytidine (AZA), 5-aza-2′-deoxycytidine (DAC), and GSK-3685032 (GSK5032) for 3 and 7 days.
Supplementary Table 6. Average coverage per CpG for low pass whole methylome sequencing of ALL-SIL, LOUCY, JURKAT, and SUP-T1 cells treated with increasing concentrations of 5-azacytidine (AZA), 5-aza-2′-deoxycytidine (DAC), and GSK-3685032 (GSK5032) for 3 and 7 days.
Supplementary Table 7. Sequencing metrics for Enzymatic Methyl-sequencing, deep sequencing, of LOUCY and SUP-T1 cells treated with 10 nM 5-aza-2′-deoxycytidine (DAC) for 3 days or 300 nM GSK-3685032 (GSK5032) for 3 and 7 days.
Supplementary Table 8. Differential methylation at promoters for LOUCY (L) and SUP-T1 (S) cells treated with 10 nM 5-aza-2′-deoxycytidine (DAC) for 3 days (D3) or 300 nM GSK-3685032 (GSK5032) for 3 (G3) and 7 days (G7). DNA methylation on a scale from 0 to 1. q value, BH-corrected p-value.
Supplementary Table 9. List of genes whose promoters show ≥ 80% DNA methylation in control cells (ctrl) or in cells treated with 300 nM GSK-3685032 (GSK5032) for 7 days (retain) in LOUCY and SUP-T1 cells.
Supplementary Table 10. Gene ontology (GO) enrichment analysis for genes retaining DNA methylation at promoters in both LOUCY and SUP-T1 cells (overlap) and for methylation-sensitive genes in LOUCY and SUP-T1 cell individually after treatment with GSK-3685032 (GSK5032) for 7 days.
Supplementary Table 11. Kyoto Encyclopaedia of Genes and Genomes (KEGG) enrichment analysis for genes retaining DNA methylation at promoters in both LOUCY and SUP-T1 cells (overlap) and for methylation-sensitive genes in LOUCY and SUP-T1 cell individually after treatment with GSK-3685032 (GSK5032) for 7 days.
Supplementary Table 12. Differential gene expression promoters for LOUCY (L) and SUP-T1 (S) cells treated with 10 nM 5-aza-2′-deoxycytidine for 3 days (D3) or 300 nM GSK-3685032 (GSK5032) for 3 (G3) and 7 days (G7).
Supplementary Table 13. Differential methylation at promoters and corresponding gene expression for promoters for LOUCY (L) and SUP-T1 (S) cells treated with 10 nM 5-aza-2′-deoxycytidine (DAC) for 3 days (D3) or 300 nM GSK-3685032 (GSK5032) for 3 (G3) and 7 days (G7). Selected for curated, NM_* and NR_*, transcripts.
Supplementary Table 14. Genes used for enrichment analyses.
Supplementary Table 15. Differential methylation of transposable elements for LOUCY cells (L) treated with 10 nM 5-aza-2′-deoxycytidine (DAC) for 3 days (D3) or 300 nM GSK-3685032 (GSK5032) for 3 (G3) and 7 days (G7).
Supplementary Table 16. Differential methylation of transposable elements for SUP-T1 cells (S) treated with 10 nM 5-aza-2′-deoxycytidine (DAC) for 3 days (D3) or 300 nM GSK-3685032 (GSK5032) for 3 (G3) and 7 days (G7).
Supplementary Table 17. Differential expression of transposable elements for LOUCY cells (L) treated with 10 nM 5-aza-2′-deoxycytidine (DAC) for 3 days (D3) or 300 nM GSK-3685032 (GSK5032) for 3 (G3) and 7 days (G7).
Supplementary Table 18. Differential expression of transposable elements for SUP-T1 cells (S) treated with 10 nM 5-aza-2′-deoxycytidine (DAC) for 3 days (D3) or 300 nM GSK-3685032 (GSK5032) for 3 (G3) and 7 days (G7).
Supplementary Table 19. Genes used for the enrichment analysis of the molecular signature database datasets HALLMARK_INTERFERON_ALPHA_RESPONSE (M5911) and the CP:REACTOME dataset REACTOME_STING_MEDIATED_INDUCTION_OF_HOST_IMMUNE_RESPONSES (M27045), along with whether they were detected and differentially expressed in LOUCY and SUP-T1 cells treated with 300 nM GSK-3685032 for 7 days (G7).
Supplementary Table 20. Overview of nucleoside and non-nucleoside compounds.

Additional file 3. Complete raw images for western blots shown in Supplementary Fig. 6A-C.

## Data Availability

All the data generated in this paper is available at ArrayExpress (RRID:SCR_002964) under accession numbers: E-MTAB-14375 (total RNA sequencing data), E-MTAB-14202 (high coverage Enzymatic Methyl-sequencing) and E-MTAB-14197 (low-coverage whole methylome sequencing). All other data supporting the findings of this study are available from the corresponding author upon reasonable request.

## Code availability

All code used for data analysis is available at https://github.com/ColmNestorLab/HMA_TALL.
