# Supplementary material for: Exposing the DNA methylation-responsive compartment of the leukaemic genome in T-ALL cell lines support its potential as a novel therapeutic target in T-ALL
Source: Clin Epigenetics. 2025 Jul 3;17:114. doi: 10.1186/s13148-025-01915-y (PMC12224799; doi:10.1186/s13148-025-01915-y)

Supplementary Figure 1

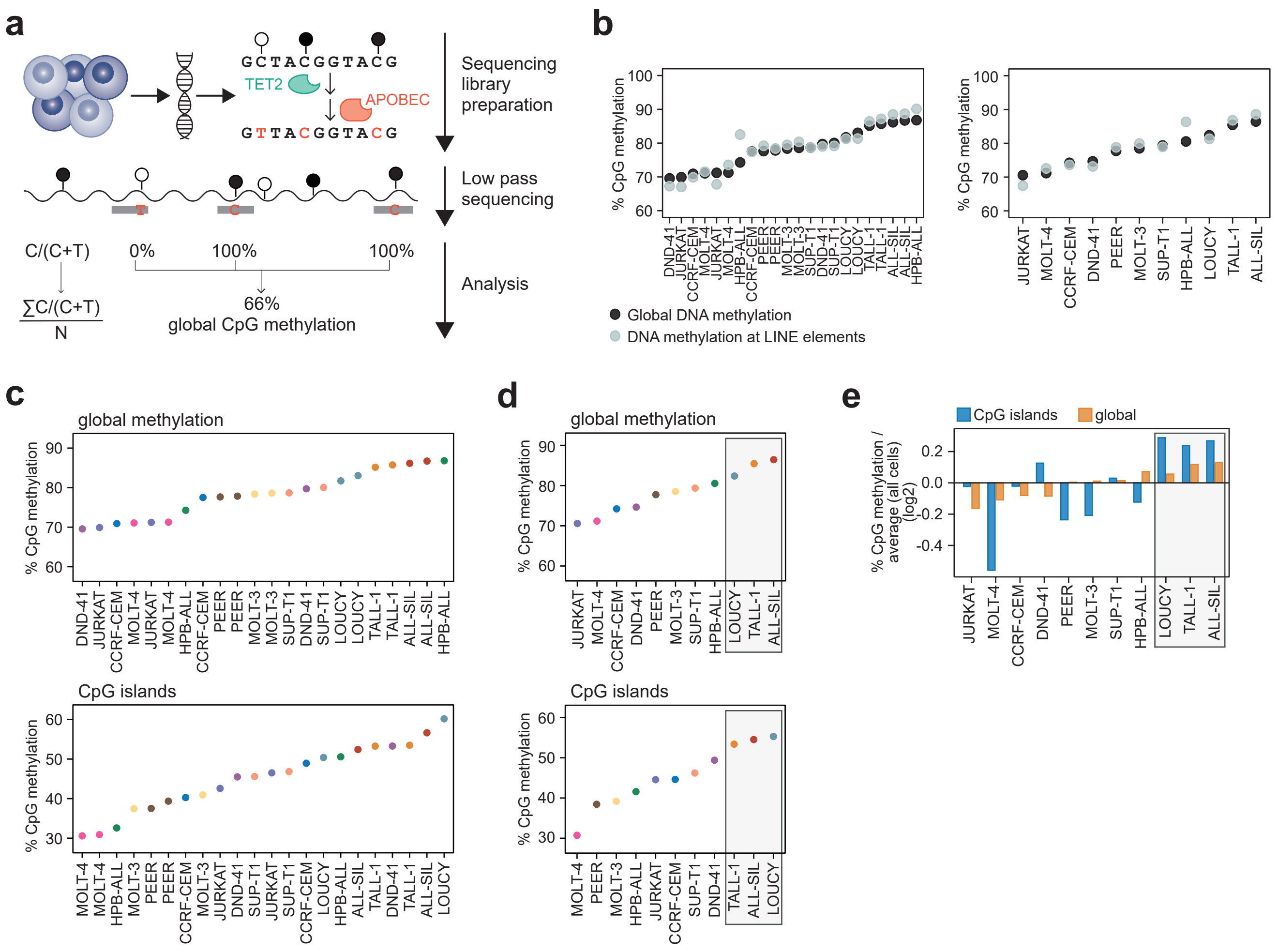

Supplementary Figure 2

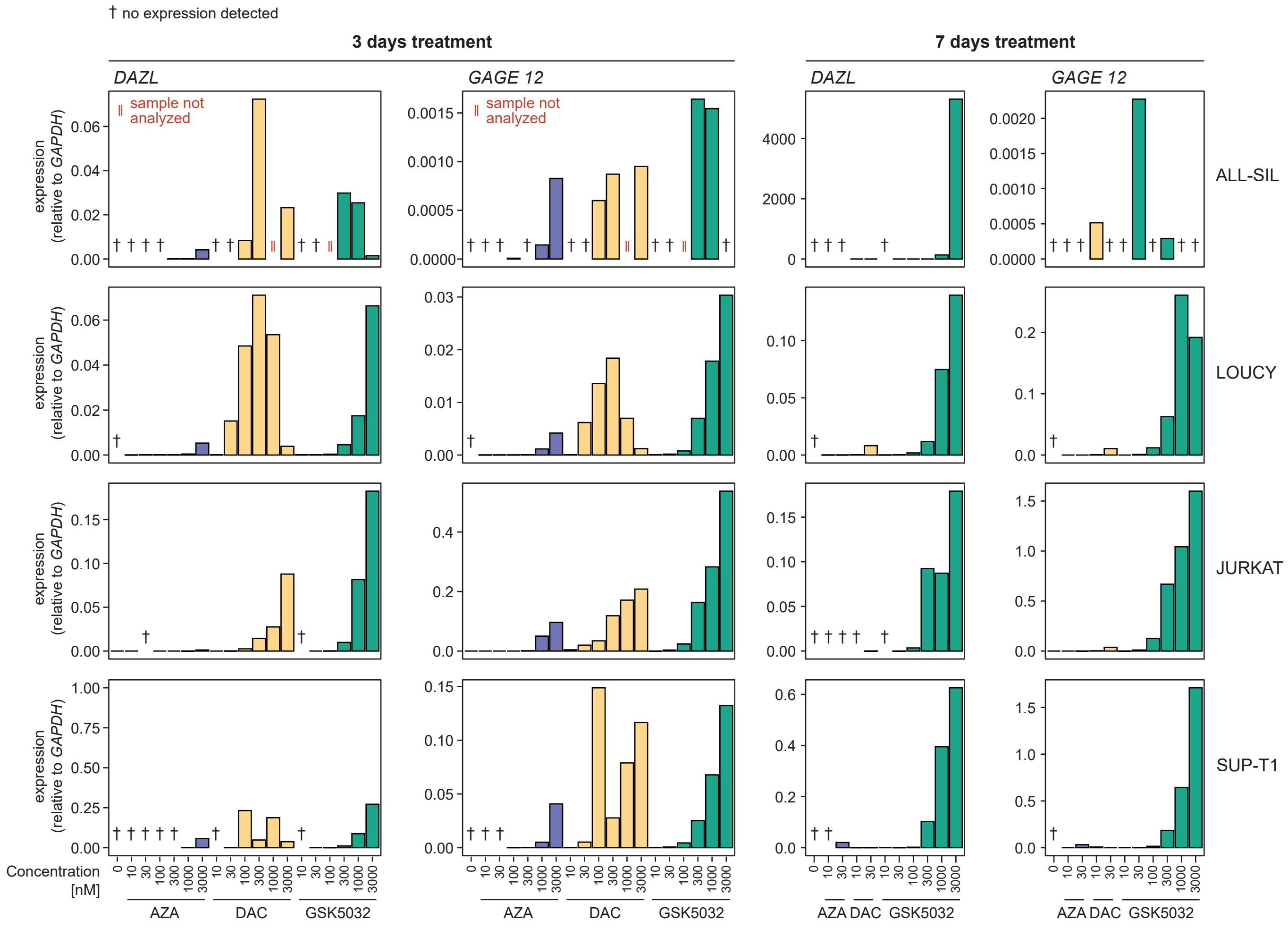

Supplementary Figure 3

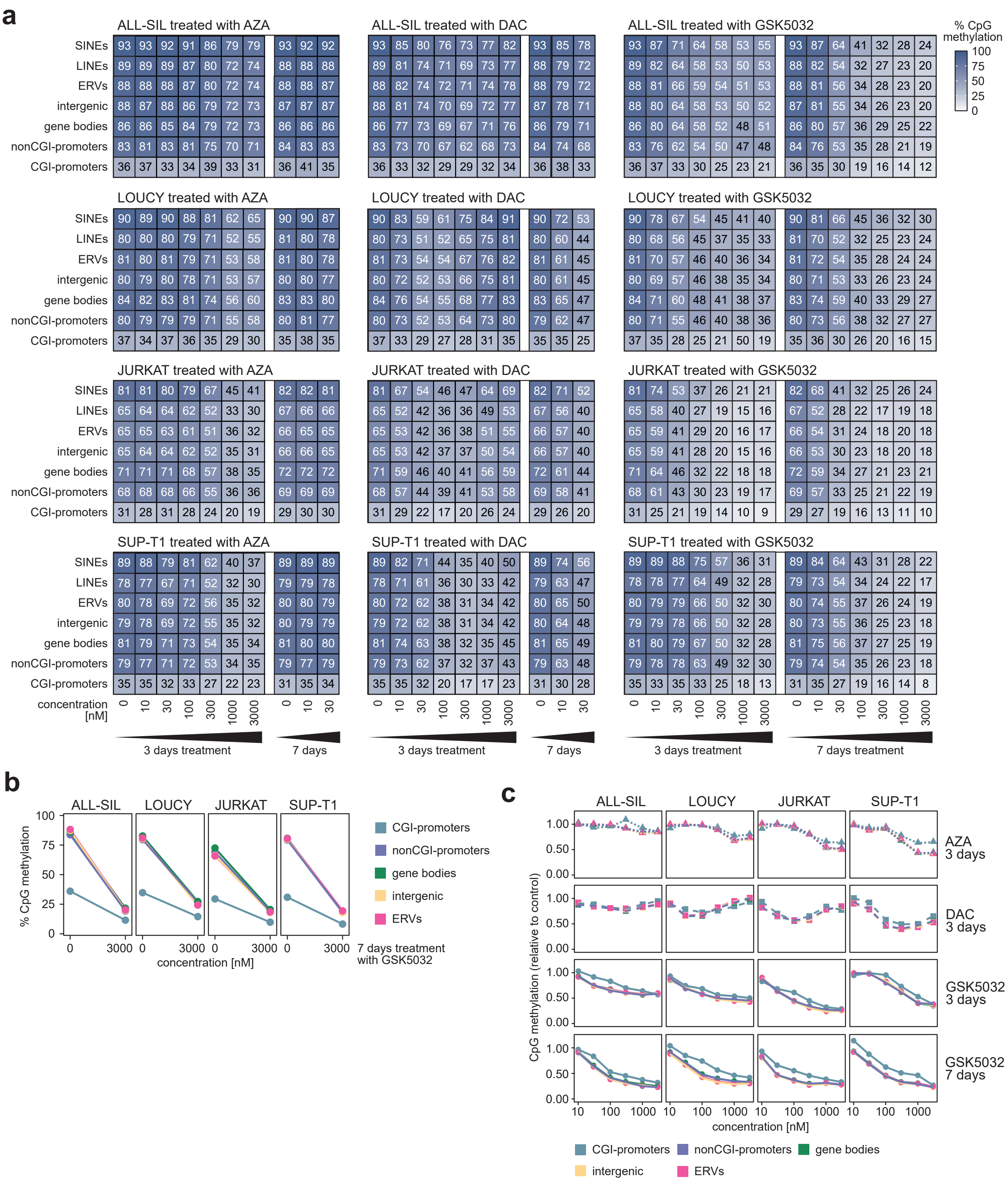

Supplementary Figure 4

a

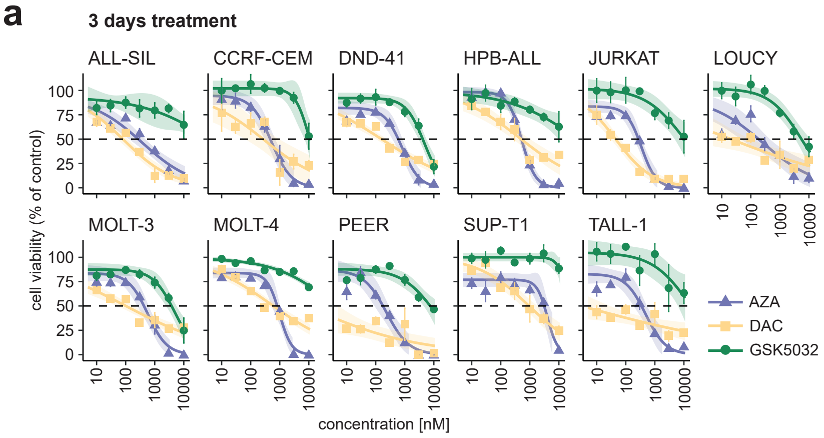

b

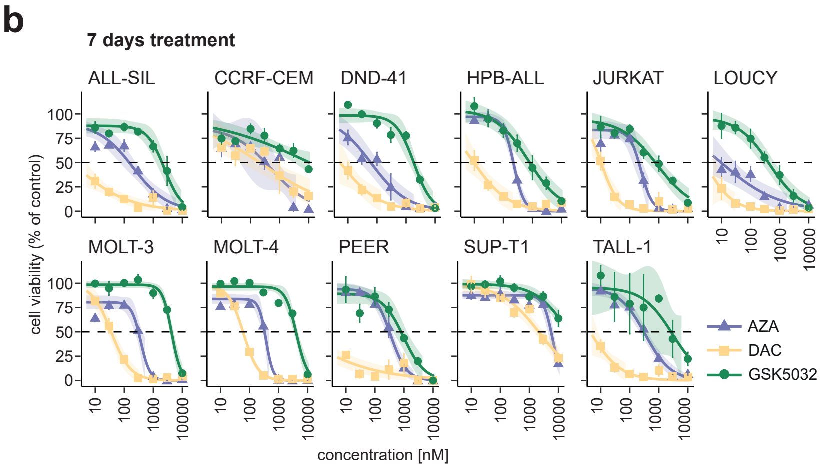

Supplementary Figure 5

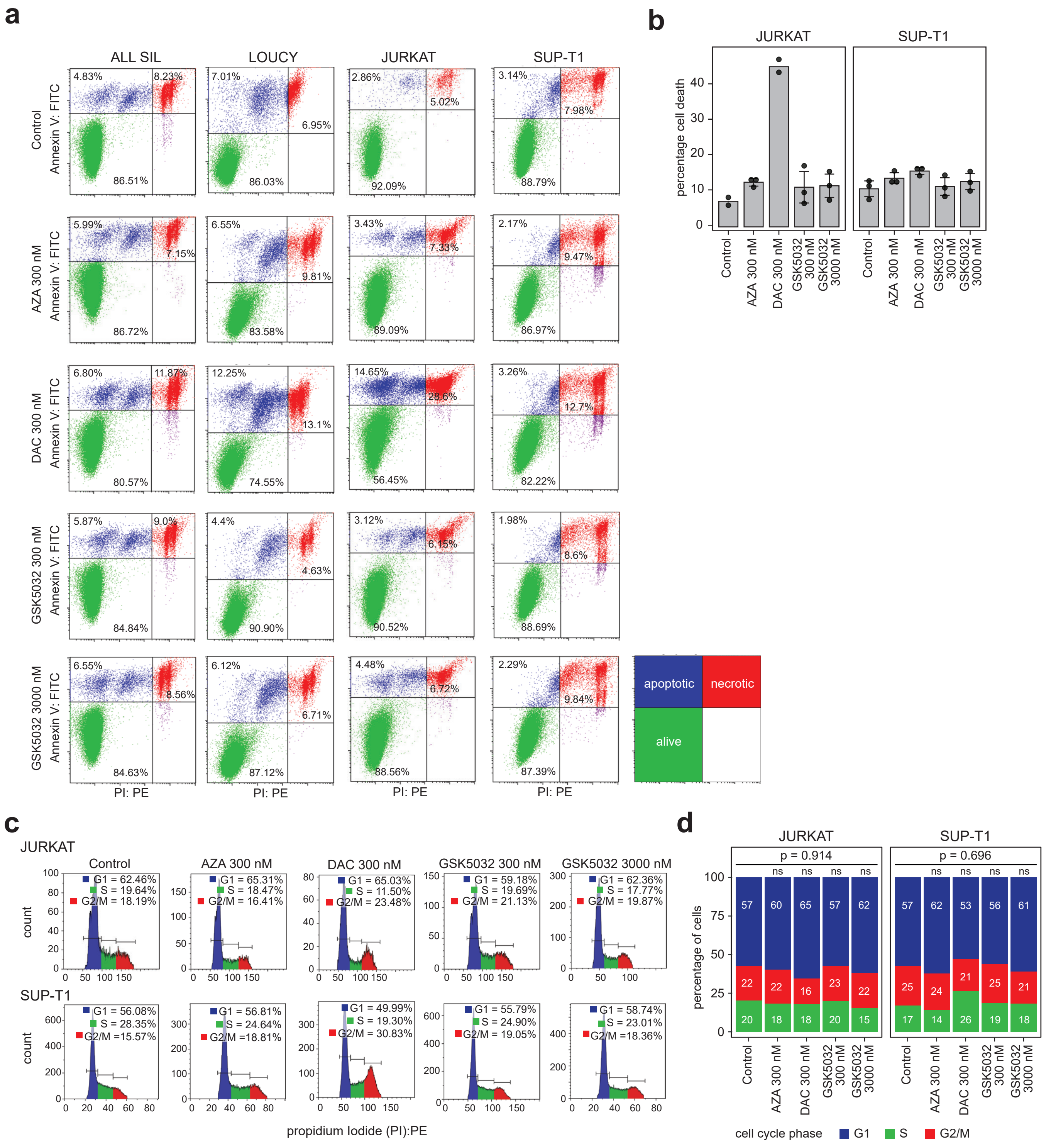

Supplementary Figure 6

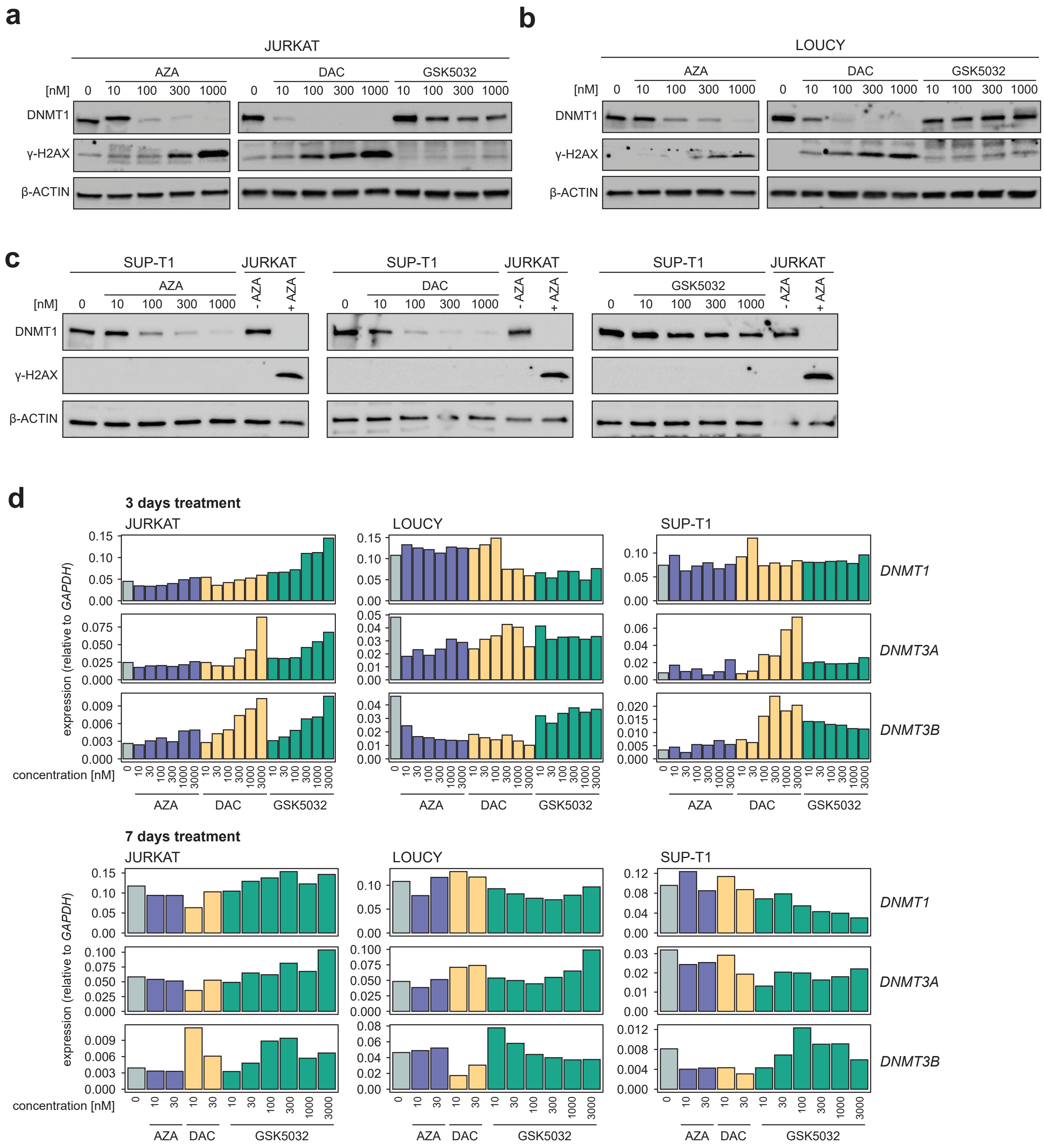

Supplementary Figure 7

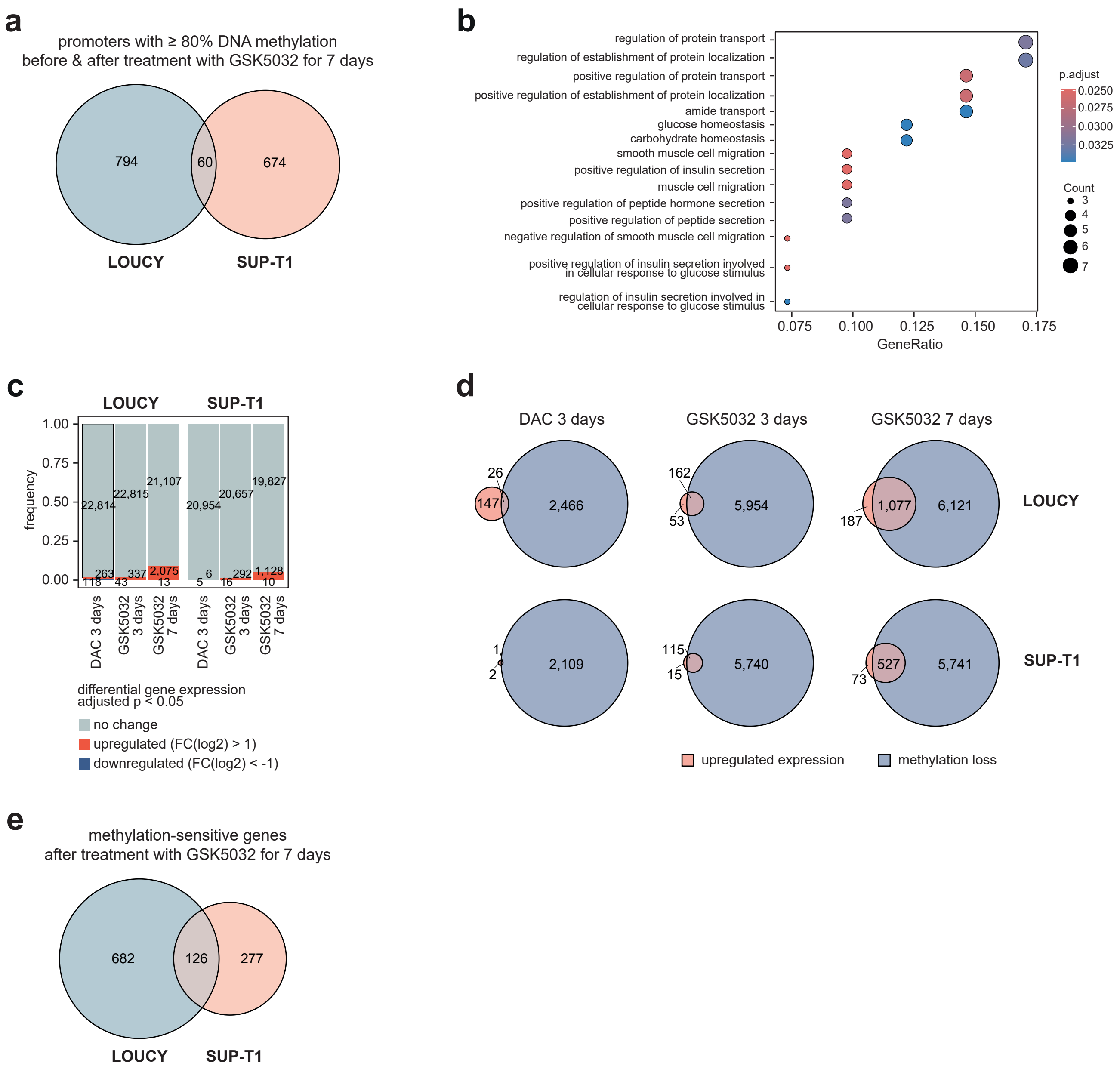

Supplementary Figure 8

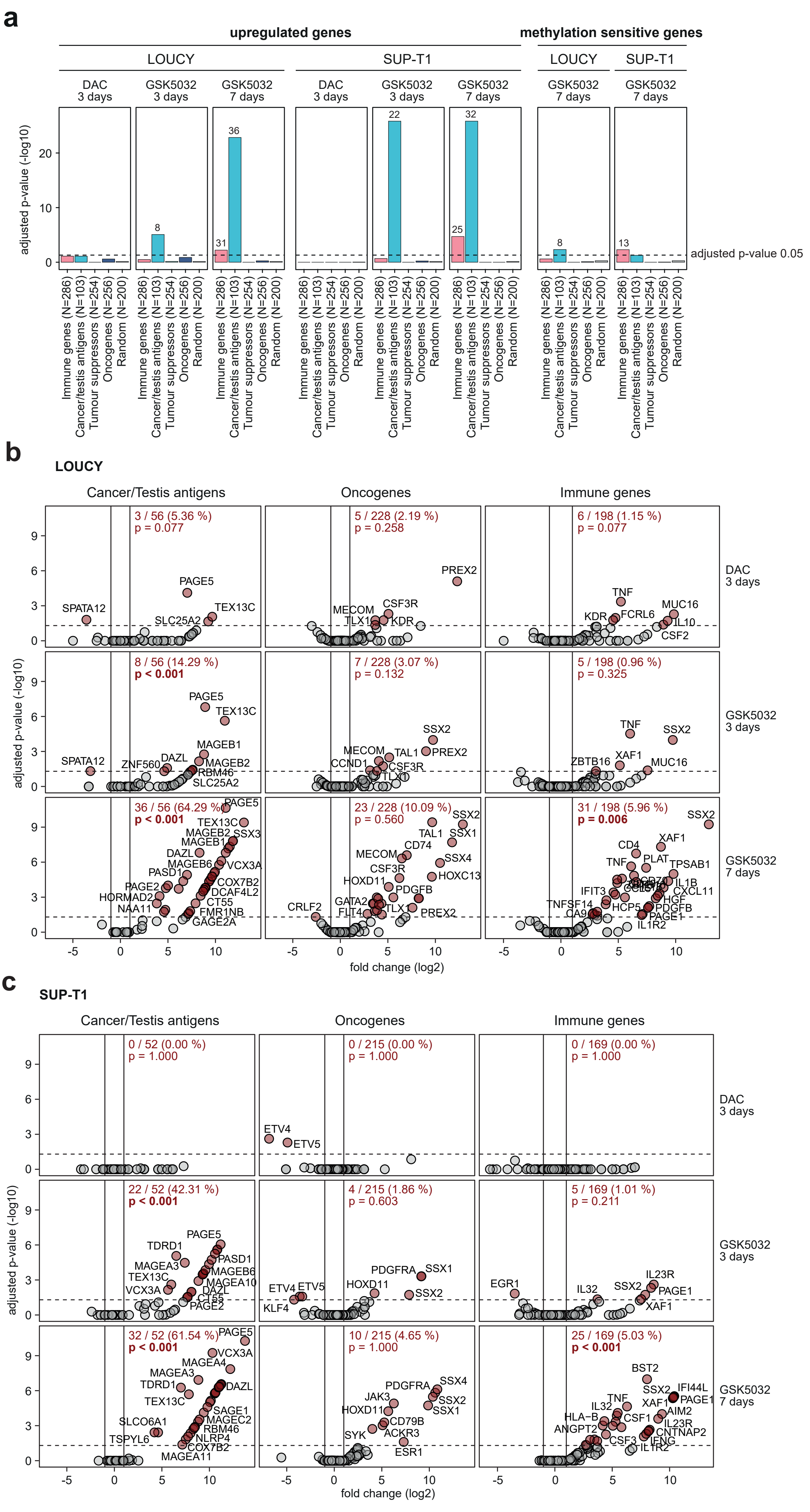

Supplementary Figure 9

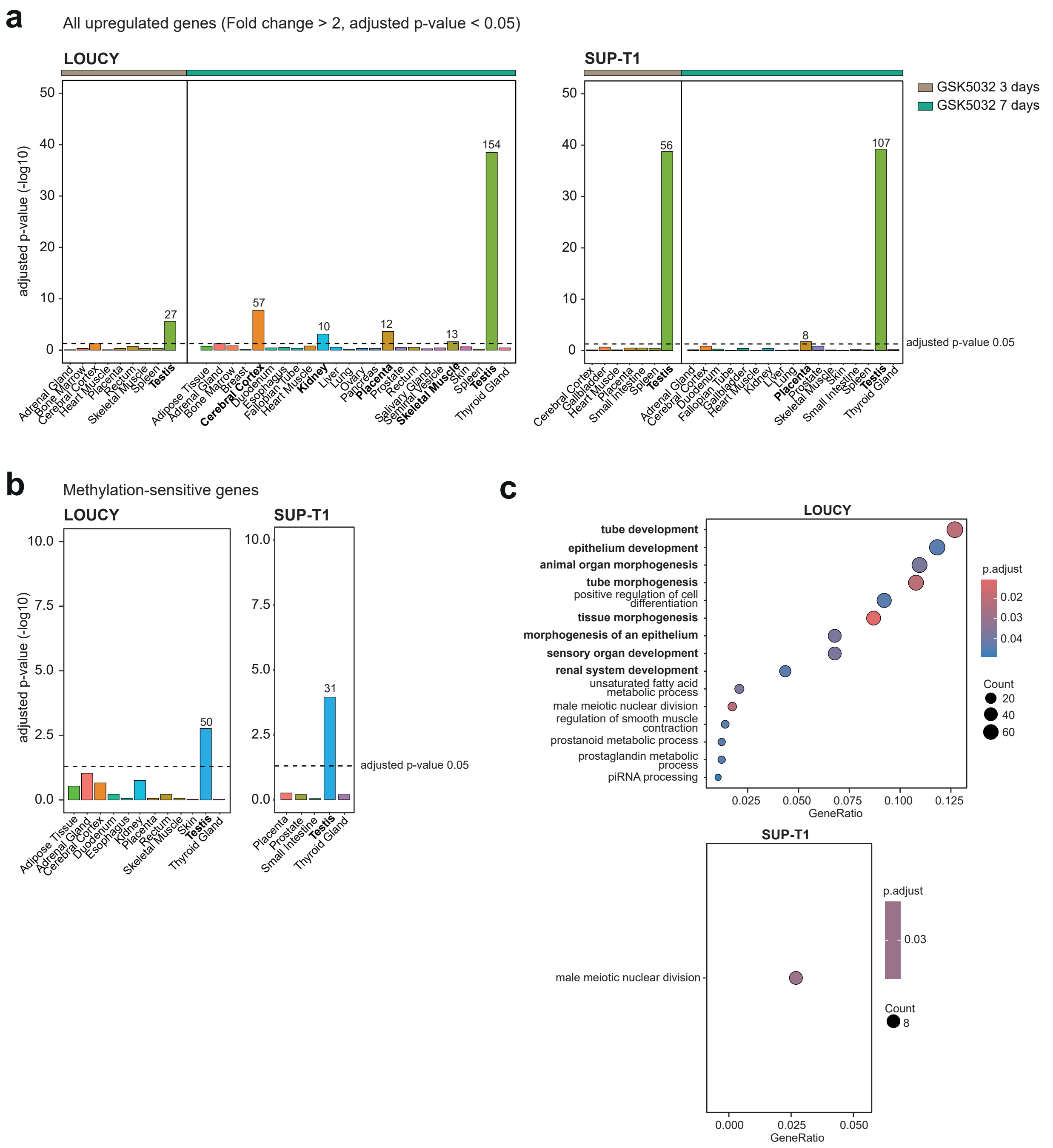

Supplementary Figure 10

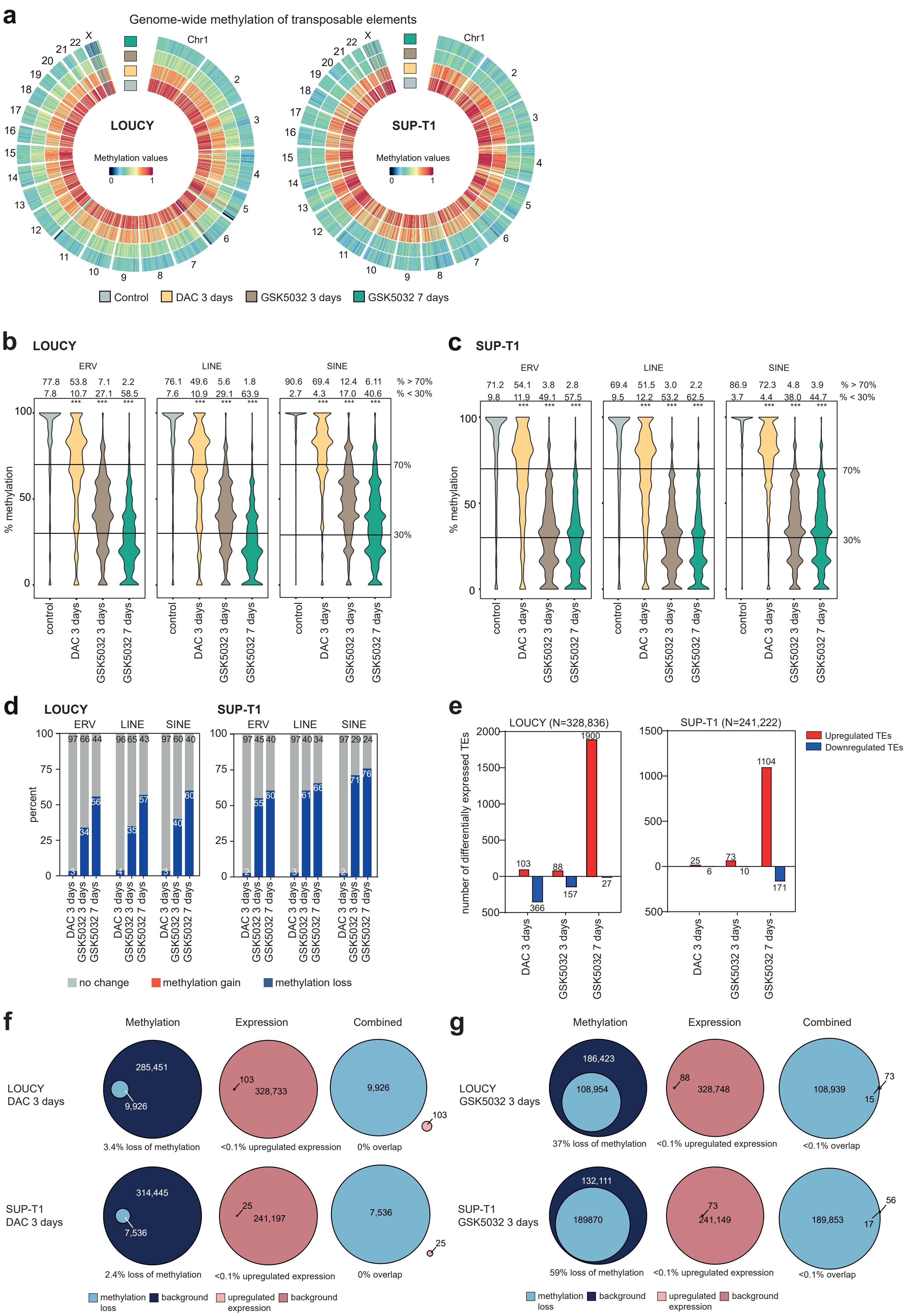

Supplement: Supplementary file 1 — Additional file 1. Supplementary Fig. 1.a, Overview of our low-coverage whole methylome sequencing approach. DNA was extracted and base pair resolution DNA methylation libraries were prepared by enzymatic methyl-conversion using TET2 and APOBEC. Sequencing libraries were sequenced at low coverage and DNA methylation per covered CpG was assessed from the number of sequenced unconverted (methylated) cytosines (C) and the number of sequenced thymines (T) representing unmethylated cytosine. Global or region-specific DNA methylation was determined by calculating the average across all covered CpGs.b, Average global CpG methylation assessed by low-coverage whole methylome sequencing (black) and DNA methylation at long interspersed nucleotide elements (LINE) (grey) for T-ALL cell lines. Individual points for each replicate (left) and mean of duplicates (right).c,d Average CpG methylation as percentage genome-wide (global) or at CpG islands for 11 T-ALL cell lines assessed by low-coverage whole methylome sequencing. Individual replicates (c) and mean of duplicates (d). e, Percentage of CpG methylation relative to the average over all cell lines for CpG islands and globally. . Supplementary Fig. 2. Expression of genes known to get re-expressed upon DNA demethylation, DAZL, and GAGE12, after treatment with increasing concentrations of 5-azacytidine (AZA), 5-aza-2′-deoxycytidine (DAC), and GSK-3685032 (GSK5032) for 3 (left) and 7 days (right). Expression determined by qPCR and shown relative to housekeeping gene GAPDH. Cross indicates that no expression was detected by qPCR. Supplementary Fig. 3.a, Average global CpG methylation relative to an untreated control across four T-ALL cell lines divided based on genomic location. Cells were treated with increasing concentrations of 5-azacytidine (AZA), 5-aza-2′-deoxycytidine (DAC), and GSK-3685032 (GSK5032) for 3 or 7 days. b, Average absolute CpG methylation across four T-ALL cell lines treated with 3000 nM GSK5032 for 7 days divid [file 13148_2025_1915_MOESM1_ESM.pdf]
